# 5‐Hydroxytryptamine Distribution Alteration in Both Neuron and Synapse of Tg(SOD1*G93A)1gur Mice: A Potential Intervention Candidate Strategy for Amyotrophic Lateral Sclerosis

**DOI:** 10.1002/cns.70946

**Published:** 2026-05-29

**Authors:** Lijun Zhou, Menghua Li, Qi Dai, Xiwang Liu, Cheng Li, Huifeng Jiao, Haili Pan, Renshi Xu

**Affiliations:** ^1^ Jiangxi Medical College Nanchang University Nanchang China; ^2^ Department of Neurology Jiangxi Provincial People's Hospital, the First Affiliated Hospital of Nanchang Medical College, National Regional Medical Center for Nervous Disease Nanchang Jiangxi China; ^3^ Department of Neurology First Affiliated Hospital of Nanchang University Nanchang Jiangxi China; ^4^ Institute of Life Science Nanchang University Nanchang Jiangxi China; ^5^ Basic Medical School Nanchang University Nanchang Jiangxi China

**Keywords:** 5‐hydroxytryptamine, amyotrophic lateral sclerosis, brainstem, pathogenesis, spinal cord

## Abstract

**Aims:**

Amyotrophic lateral sclerosis (ALS) is a fatal neurodegenerative disease; the precise pathogenesis of sporadic ALS (sALS) has not yet been elucidated up to now. Previous studies revealed that the abnormal alterations of some non‐motor neurons (non‐MN) were a potential pathogenesis of sALS. Therefore, this study aims to search the potential evidences of non‐MN in the pathogenesis of ALS via exploring potential relationships between 5‐hydroxytryptamine (5‐HT) neurons and the development of ALS.

**Methods:**

We employed fluorescent immunohistochemistry to investigate the altered distribution patterns of 5‐HT and tryptophan hydroxylase 2 in the spinal cord and brainstem of Tg(SOD1*G93A)1Gur (TG) and wild‐type (WT) mice. Additionally, we used western blot to analyze the expression levels of 5‐hydroxytryptamine receptor 1A (5‐HTR1A) and 5‐HTR2A.

**Results:**

Our findings revealed that 5‐HT synapses were primarily distributed in the funiculus lateralis, anterior horn, posterior horn, central lateral column, and the area around the central canal of cervical, thoracic, and lumbar segments, and raphe nucleus as well as lateral paragigantocellular nucleus, and gradually reduced following age increase in WT mice. However, 5‐HT synapses in the spinal cord and 5‐HT neurons in the brainstem gradually increased following the progression of disease and presented a significantly negative correlation between the increased distribution of 5‐HT synapses and neurons and the reduction of neural cell number (positively correlated with the increase in neural cell death) at the onset and/or progression stage of TG mice. 5‐HTR1A significantly increased, while 5‐HTR2A significantly decreased at the onset stage of TG mice.

**Conclusion:**

Our study speculated that the distribution changes of 5‐HT synapses in the spinal cord and 5‐HT neurons in the brainstem play a potential protective role in the pathogenesis of sALS through a compensatory 5‐HT increase.

## Graphical abstract

This study reveals that 5‐hydroxytryptamine (5‐HT) synapses in the spinal cord and 5‐HT neurons in the brainstem of the SOD1‐G93A amyotrophic lateral sclerosis (ALS) mouse model are significantly altered, showing a decrease in pre‐symptomatic stages but a marked increase as the disease progresses. This increase in 5‐HT synaptic and neuronal distribution correlates negatively with motor neuron death. The findings suggest that the dysregulation of 5‐HT systems is closely associated with neurodegeneration, identifying it as a potential pathogenesis and therapeutic strategy in ALS.

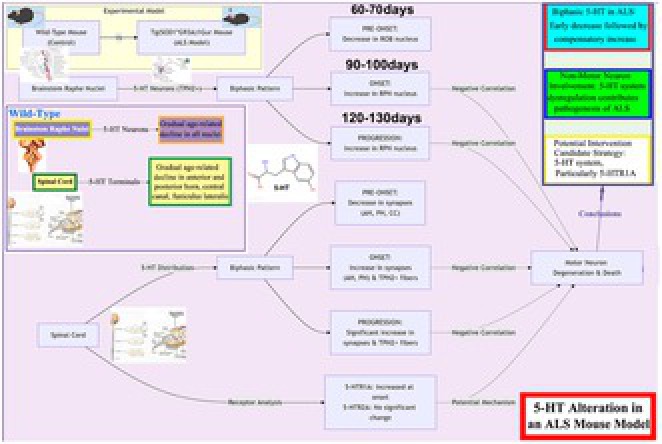

## 1 Introduction

Amyotrophic lateral sclerosis (ALS) is a neurodegenerative disease belonging to motor neuron (MN) diseases; the worldwide prevalence of ALS is 4–6 in 100,000 populations; there is significant differences among different populations [1, 2]. Approximately 10% of ALS patients are the familial type; familial ALS (fALS) mostly exhibits an autosomal dominant inheritance pattern; 90% of patients have not the exact background of genetics, known as sporadic ALS (sALS) [3]. The average onset age of sALS is 60 ± 5 years, but the onset age of fALS is earlier than that of sALS [4]. The average disease course of sALS is about 4.4 years from the onset of diagnosis; approximately 25% of sALS patients have a survival time exceeding 5 years; and the survival time of 5%–10% sALS cases exceeds 10 years from the begin of diagnosis [5]. ALS patients usually die from respiratory failure [4, 5, 6]. ALS is mainly featured by the selective and progressive degeneration of both superior and inferior MN involving the cerebrum, brainstem as well as spinal cord, which results in the muscle atrophy of laryngopharyngeal, limbs, and even whole body and gradually paralyzes [3].

To date, the pathogenesis of sALS remains incompletely understood. Based on current research findings, it is suggested that the following potential pathogenesis may be closely related to the degeneration of MN in sALS, which involve in the toxic of mutative ribonucleic acid, the excitatory toxicity, the disorder of protein balance, the defection of axon transportation, the excessive production of reactive oxygen species, the lesion of mitochondria function, and the abnormal alteration of non‐MN [6, 7, 8, 9, 10, 11].

We hypothesized that sALS should not be the only sole neurodegeneration and the death of MN but it might also affect other neural cells besides MN according to the currently studied reports about the pathogenesis of sALS [6, 8, 9, 10, 11]. The currently researched evidence demonstrates that the functional dysregulation between glia and MN also plays an important role in the pathogenesis of sALS; moreover, the increased damage vulnerability in distal axonal compartments is observed at the early stages of sALS patients, which are consistent with the current insight that the lesion of MN in sALS is resulted by the interaction effects of multiple and complex pathophysiological processes [6, 7, 8, 9, 10, 11, 12]. It is thought that sALS only the alone damaged MN in the traditional viewpoint. In fact, more and more evidences have proved that other nervous systems besides motor systems are also involved in the pathogenesis of sALS at present. Therefore, we hypothesized that the pathogenesis of sALS might be similar to that of both Alzheimer's disease and Parkinson's disease; it was not only to damage the hippocampus neurons of memory function in Alzheimer's disease and the dopaminergic neurons of the midbrain in Parkinson's disease, respectively, but also damage other neural cells. Current evidence has demonstrated that both Alzheimer's disease and Parkinson's disease extensively damage various neurons, in addition to hippocampus memory neurons and dopaminergic neurons, and even involve glial cells. Therefore, we hypothesize that sALS may share common or similar pathogenesis with both Alzheimer's disease and Parkinson's disease and also involve damage to multiple neurons or glial cells.

The staging pattern of sALS based on the pathological alteration of phosphorylated TAR deoxyribonucleic acid‐binding protein‐43 (pTDP‐43) consists of four stages. pTDP‐43 inclusion bodies are found in the non‐granular motor cortex and the α motor neurons of the brainstem and spinal cord, a condition known as stage 1. pTDP‐43 in the prefrontal neocortex, the reticular formation, and the pre‐cerebellar nuclei is known as stage 2; pTDP‐43 surrounding the regions of prefrontal neocortex and in the postcentral location of sensory cortex and basal ganglia is known as stage 3; pTDP‐43 occurs in the anteromedial temporal lobe including hippocampus is known as stage 4. According to this staging pattern, the corticofugal axonal pattern of pathological spread is speculated, by which the pathogenesis of sALS begins in the primary motor cortex and extends from the primary motor cortex to the inferior MN and to the sub‐cortical structure by the axon projection. Recent neuroradiological evidence supports adherence to this staging method. According to clinical observations, sALS has been found to partially involve non‐motor deficits, which appear to be pathologically responsible for involving other related non‐motor brain structures [13, 14, 15, 16, 17]. The staging pattern of sALS based on the spread of pTDP‐43 further fully proves that the sALS pathological lesion in nervous systems is far beyond the motor areas in the cerebral cortex, brainstem, and the anterior horn of spinal cord.

The investigator recently proposed four different disease periods of sALS based on the distribution patterns of pTDP‐43 intraneuronal inclusions [18]. It is theoretically notable that, in sALS, the regions projecting to the cortex are damaged, and this damage is distinct from the regions of corticofugal axon projections. This distinction supports the hypothesis that pTDP‐43 may spread in a prion‐like propagating manner, potentially originating from the motor cortex and spreading downward. It is consistent with the above insight that superior raphe nuclei (RN) that cortex diffusely projects almost are not involved by pathological pTDP‐43 in sALS, avoiding the lesion involvement of pathological pTDP‐43, which is in almost agreement with the α synuclein spread form of Parkinson's disease [19]. The RN is a major anatomical region for 5‐hydroxytryptamine (serotonin, 5‐HT) neuron distribution, with approximately 80% of 5‐HT neurons in the central nervous system (CNS) located in the RN. Therefore, we hypothesized that the related molecules of 5‐HT neuron functions such as 5‐HT1A/2 receptor agonist, 5‐HT precursor 5‐hydroxytryptophan (5‐HTP) [20], or 5‐HT2B/C receptor agonist [21] may be partially protect MN functions, even significantly reverse the progression of sALS. In general, the potential effects about 5‐HT in the pathogenesis of sALS theoretically have obtained new understanding, especially because several related papers recently are published [21, 22, 23].

5‐HT is a monoamine neurotransmitter in the CNS, and it is well known that 5‐HT is an important neurotransmitter for transmitting happiness feel [24]. Approximately 90% of 5‐HT primarily distribute in the enterochromaffin cell located in the gastro‐intestine tracts besides CNS [25]. The remainder of 5‐HT is synthesized in the 5‐HT neurons of the CNS, with the majority being distributed in the RN of the brainstem, where it exerts important physiological functions, regulating mood, appetite, and sleep. In addition, 5‐HT also plays roles in cognitive functions such as memory and learning.

The RN neuron is the major source synthesizing and releasing 5‐HT neurotransmitter in CNS [26], it consists of nine RNs including B1‐9 nuclei. RNs constitute the major part of 5‐HTergic neurons. Sometimes, the clusters of RNs are linearly regarded as a single nucleus. Nine RNs distribute linearly in the midline of the brainstem surrounding the reticular formation of brainstem [27]. Axons projecting from RN neurons to other neural structures form the 5‐HTergic neurotransmitter systems, reaching nearly all parts of the CNS. 5‐HTergic neuronal axons in the inferior RNs terminate among the cerebellum and spinal cord, but the 5‐HTergic axons from the upper RN nucleus almost project to the entire brain.

Although current investigation evidence indicates that the 5‐HT neurotransmitter may be involved in the development of sALS and play certain roles in sALS, the precise mechanisms and effects of the 5‐HT neurotransmitter on sALS have not been fully clarified, and there is still some debate regarding this issue at present. To this end, we observed and analyzed the altered features of 5‐HT distribution in neurons and synapses in the spinal cord and brainstem of mainly damaged regions in sALS, and the relationship between the 5‐HT alteration and the death of neural cells applying both Tg(SOD1*G93A)1Gur (TG) and wild‐type (WT) mice. Our findings indicate a significant elevation of 5‐HT neurotransmitter levels in the spinal cord and brainstem of Tg(SOD1*G93A)1Gur mice, which correlates with the observed neural cell death in these transgenic animals. Our study suggested that the 5‐HT neurotransmitter in the neuron and synapse of both spinal cord and brainstem might be the potential pathogenesis and intervention candidate strategy of neuronal death in the development of sALS.

## 2 Materials and Methods

### 2.1 G93A‐SOD1 Mice

The TG mice from Jackson Laboratory (cat#002726, Jackson Laboratory, Bar Harbor, ME, USA) were bred by mating male TG mice with female WT mice (cat#000664, Jackson Laboratory, Bar Harbor, ME, USA) at The First Affiliated Hospital of Nanchang University. The experimental mice were isolated genome deoxyribonucleic acid (DNA) from mice tail tissue by the Rapid Animal Genomic DNA Isolation Kit (cat#B518221, Sangon Biotech, Shanghai, China). The TG mice were identified whether or not the G93A‐positive TG mice by the polymerase chain reaction (PCR) of genome DNA from the mice tail tissue. The used primers (Sangon Biotech, Shanghai, China) in PCR were as follows: the forward primer of IL‐2 (PCR internal reference) was 5′‐CTA GGC CAC AGA ATT GAA AGA TCT‐3′, the reverse primer of IL‐2 was 5′‐GTA GGT GGA AAT TCT AGC ATC C‐3′, the forward primer of hmSOD1 G93A was 5′‐CAT CAG CCC TAA TCC ATC TGA‐3′, and the reverse primer of hmSOD1 G93A was 5′‐CGC GAC TAA CAA TCA AAG TGA‐3′. The PCR protocol specified: denaturation at 94°C for 3 s, annealing at 60°C for 1 min, and extension at 72°C for 1 min, repeated for 35 cycles. All experimental mice used in this study were male. Animals were sacrificed at three time points: pre‐onset (60–70 days), onset (90–100 days), and progression (120–130 days). Each mouse was euthanized with CO_2_ euthanasia. The severity of hind limb muscle atrophy was determined by hematoxylin eosin staining of the hind limb gastrocnemius muscles at different disease stages, and the different disease stages were evaluated histologically by observing the changes in muscle structure under light microscopy [28, 29, 30, 31].

The experimental mice were housed under 20°C–27°C, 40%–50% humidity, a 12‐h light/dark cycle, and free access to food or water. The ALS Therapy Development Institute (ALSTDI) score was used to monitor health and behavioral disorders in mice [32, 33]. ALSTDI score was used to further determine the disease courses and stages. Experiments were designed in such a way that total number of animals used and suffering were minimized. All mice used in this study were manipulated the Chinese Guide for the Care and Use of Laboratory Animals and were reviewed by the Animal Care and Use Ethics Committee.

### 2.2 Fluorescence Immunohistochemistry Staining of Both Spinal Cord and Brainstem

Both brain and spinal cord were dissected following adequate perfusion, approximately 20 mL of 0.9% saline and 40 mL of 4% 1xPBS pH 7.5 paraformaldehyde solution (cat#P7059, Sigma‐Aldrich, St. Louis, MO, USA) via the right ventricle at room temperature (RT) after anesthesia induction with CO_2_. The detailed protocol was similar to the experimental methods of Zhou et al. [31] Both spinal cord and brain were immediately put into 4% paraformaldehyde (cat#F8775, Sigma‐Aldrich, St. Louis, MO, USA) overnight after taken out, then transferred to a 20% sucrose solution in 1xPBS (pH 7.5) for incubation, followed by embedding in optimal cutting temperature compound (OCT, cat#4583, Sakura Finetek, Chuo‐ku, Tokyo, Japan). The spinal cord and brainstem tissues were sequentially sectioned into 12 μm coronal slices using a freezing microtome, and the slices were placed onto Superfrost Plus slides. The fluorescence immunohistochemistry staining of both spinal cord and brainstem was conducted by the following processes; section was permeabilized with 0.2% TritonX‐100 (cat#T8787, Sigma‐Aldrich, St. Louis, MO, USA) and blocked with 10% 1xPBS bovine serum albumin (cat#SRE0098, Sigma‐Aldrich, St. Louis, MO, USA) after rehydration in pH 7.4 1xPBS. The following primary antibodies were added according to the requirement of the experimental design: 1:100 rabbit anti‐vimentin (cat#EPR3776 RRID:AB_92547, Abcam, Cambridge, MA, USA), 1:100 rabbit anti‐Nestin (cat#SP103 RRID:AB_105389, Abcam, Cambridge, MA, USA), 1:200 rabbit anti‐NeuN (cat#EPR12763 RRID:AB_ab177487, Abcam, Cambridge, MA, USA), 1:1000 rabbit anti‐GFAP (cat#EPR19996 RRID:AB_ab7260, Abcam, Cambridge, MA, USA), 1:400 rat anti‐serotonin (5‐HT) (cat#YC5/45 RRID:AB_ab6336, Abcam, Cambridge, MA, USA), and 1:600 mouse anti‐TPH2 (cat#CL2990 RRID:AB_ab211528, Abcam, Cambridge, MA, USA) monoclonal antibody. All primary antibodies were incubated overnight at 4°C and then lightly washed with 0.2% 1X PBS Triton X‐100 buffer six times each 5 min. The following secondary antibodies conjugating to green fluorescence or red rhodamine were added and incubated for 2 h at RT: 1:250, donkey anti‐rabbit IgG (H + L) cross‐adsorbed secondary antibody, Alexa Fluor 488 (RRID:AB_ab150073, Abcam, Cambridge, MA, USA), 1:250, donkey anti‐rat IgG (H + L) cross‐adsorbed second antibody, Alexa Fluor 488 (RRID:AB_ab150153, Abcam, Cambridge, MA, USA), and 1:200 donkey anti‐mouse IgG (H + L) cross‐adsorbed second antibody, Alexa Fluor 488 (RRID:AB_ab150105, Abcam, Cambridge, MA, USA), followed by DAPI (blue) staining and anti‐fluorescence quenching treatment after gently washing six times, with each wash lasting 5 min. All slices were detected by a fluorescence microscope (Nikon E800) equipped with a digital camera (Nikon, Sumida‐ku, Tokyo, Japan) and Photoshop software (Adobe Systems, San Francisco, CA, USA), selectively captured images. Fluorescence microscope exposure time and gain settings were kept consistent across all samples for each respective antibody stain to ensure comparability of fluorescence intensity between sections. Briefly, fluorescence images were captured using a Nikon E800 microscope equipped with a digital camera. To ensure consistency across all samples, imaging parameters were first optimized for each fluorescent channel. For the Alexa Fluor 488‐conjugated antibodies (targeting Vimentin, Nestin, NeuN, GFAP, 5‐HT, and TPH2) and DAPI nuclear stain, exposure time and gain were adjusted on representative control sections to achieve a strong signal‐to‐noise ratio while ensuring no pixel saturation. Once established, these acquisition settings were maintained constant for imaging all spinal cord and brainstem sections within the same experimental cohort. The double labeled fluorescence histochemical stain was performed by conjugating to Vimentin, Nestin, NeuN, GFAP, 5‐HT, and TPH2 antibody and DAPI to identify the proliferation and the cellular types of 5‐HT neurons.

### 2.3 Analysis of 5‐HT and TPH2 Distribution and Positive Cells

The analysis of 5‐HT and TPH2 distribution and positive cells was conducted as follows: We counted the number of 5‐HT and TPH2 stripe or linear distributions in the positive cells of both the spinal cord and brainstem from 10 sections at 200 times magnification. Then, we calculated the sum of positive cells in these 10 sections and divided the total by 10 (the total number of sections). There were five mice in each group, and 10 slices were taken from each animal. The average amount was applied to conduct a quantitation analysis. We identified and selected the interest nervous regions of cervical, thoracic, and lumbar segments of spinal cord and the interest nervous regions of brainstem based on the spinal cord and brain anatomic atlas of mice according to our previous study protocol [31, 34, 35]. Image J software (1.8.0 version) was used to quantify 5‐HT‐ and TPH2‐positive cells in immunofluorescence images. Briefly, first, defining the regions of interest (ROIs) and the objective thresholds for spinal cord and brainstem in microscopy based on a systematic approach rooted in neuroanatomy and quantitative analysis. The process began with identifying specific anatomical structures visible in microscope images, such as the dorsal and ventral horns of spinal cord or the various nuclei of the brainstem, and manually tracing their boundaries to create precise ROIs based on established anatomical landmarks. Once these regions were defined, objective thresholds were applied by establishing numerical cutoffs, such as minimum fluorescence intensity levels for positive cell identification or summed pixel values within each region, as demonstrated in techniques for measuring average fluorescence intensity using Image J to ensure that all subsequent measurements were quantitative, repeatable, and free from subjective interpretation, allowing for reliable comparison across different samples and experimental conditions. Subsequently, positive cells in ROIs were counted using Image J software (version 1.8.0).

### 2.4 Analysis of TPH2, 5HTR1A, and 5HTR2A Level in Spinal Cord by Western Blot

The temporal changes of protein levels were quantified by western blot analysis. After being quickly removed from deeply anesthetized male WT and TG mice, the spinal cords were homogenized in lysis buffer containing phenylmethylsulfonyl fluoride and protease inhibitor. The protein concentrations were then assessed by bicinchoninic acid (BCA) assays. Total protein loading was adjusted by quantifying protein concentrations using BCA assays to ensure that equal amounts (30 μg) were loaded each lane of the SDS‐PAGE gel. The protein levels of TPH2, 5HTR1A, and 5HTR2A were normalized to GAPDH, which served as an internal loading control, to account for any variations in loading or transfer efficiency. After being separated by 8% SDS‐PAGE, total proteins were transferred to polyvinylidene difluoride (PVDF) membranes. After blocked with 10% nonfat dry milk for 2 h at 4°C, the PVDF membranes were incubated with the following primary antibodies: 1:200 mouse anti‐TPH2 antibody (ab211528, Abcam Ltd., Cambridge, MA, USA), use 1:200 rabbit anti‐5HTR1A antibody (AF5453, Affinity Biosciences Ltd., Jiangshu, China), 1:200 rabbit anti‐5HTR2A antibody (bs‐12049R, Beijing Biosynthesis Biotechnology co. Ltd., Beijing, China) or 1:200 rabbit anti‐GAPDH antibody (ab245355, Abcam Ltd., Cambridge, MA, USA), and incubate at 4°C overnight. After incubation with 1:5000 horseradish peroxidase‐labeled goat anti‐mouse or goat anti‐rabbit secondary antibody (Santa Cruz Biotechnology, Santa Cruz, CA, USA) for 2 h at 4°C, the bands were visualized with enhanced chemiluminescence reagents (ECL, Pierce, Rockford, IL, USA). The protein levels were quantified and normalized to GAPDH internal controls. Three mice per group were used.

Western blot experimental conditions—Protein extraction method: Spinal cord tissues were rapidly dissected and homogenized in ice‐cold RIPA lysis buffer (50 mM Tris–HCl pH 7.4, 150 mM NaCl, 1% NP‐40, 0.5% sodium deoxycholate, 0.1% SDS) supplemented with 1 mM phenylmethylsulfonyl fluoride (PMSF) and protease inhibitor cocktail (Complete Mini, Roche Diagnostics, Mannheim, Germany). Homogenates were centrifuged at 12,000 × *g* for 20 min at 4°C, and supernatants were collected for protein concentration determination.

Protein loading amount: Protein concentrations were determined using the BCA protein assay kit (Pierce, Rockford, IL, USA) according to the manufacturer's instructions. Equal amounts of total protein (30 μg per lane) were prepared in 5× SDS loading buffer (250 mM Tris–HCl pH 6.8, 10% SDS, 30% glycerol, 5% β‐mercaptoethanol, 0.02% bromophenol blue) and heated at 95°C for 5 min before loading.

Electrophoresis conditions: Proteins were separated on 8% SDS‐polyacrylamide gels using a Mini‐PROTEAN Tetra Cell system (Bio‐Rad Laboratories, Hercules, CA, USA). Electrophoresis was performed at 80 V through the stacking gel (approximately 30 min) and then increased to 120 V for approximately 90 min until the dye front reached the bottom of the gel.

Transfer conditions: Separated proteins were transferred to PVDF membranes (Millipore, Bedford, MA, USA; pore size 0.45 μm) using a wet transfer system (Bio‐Rad). Membranes were pre‐activated in methanol for 1 min and then equilibrated in transfer buffer (25 mM Tris, 192 mM glycine, 20% methanol). Transfer was performed at 300 mA constant current for 2 h at 4°C.

Antibody incubation time: After transfer, membranes were blocked with 10% nonfat dry milk in TBST (20 mM Tris–HCl pH 7.6, 150 mM NaCl, 0.1% Tween‐20) for 2 h at 4°C. Primary antibody incubations were performed overnight (approximately 16 h) at 4°C with gentle agitation. After primary incubation, membranes were washed six times (5 min each) with TBST. Secondary antibody incubations were performed for 2 h at 4°C with gentle agitation, followed by six washes (5 min each) with TBST.

Detection method: Immunoreactive bands were visualized using ECL reagents (Pierce, Rockford, IL, USA) according to manufacturer's instructions, a technique commonly employed in western blotting to detect specific proteins. Membranes were incubated with ECL substrate for 1 min at RT and then exposed to X‐ray film (Kodak, Rochester, NY, USA) in a darkroom. To ensure signals remained within the linear range, exposure times for each antibody were meticulously calibrated, paralleling the precision required in the optimization of X‐ray machine exposure settings.

Quantification analysis method: Developed films were scanned at 600 dpi resolution using a flatbed scanner (Epson Perfection V370, Epson America Inc., Long Beach, CA, USA). Densitometric analysis was performed using ImageJ software (1.8.0 version, National Institutes of Health, Bethesda, MD, USA). After importing the western blotting image into ImageJ, the mean gray values for each band were quantified by first converting the image to an 8‐bit format, subtracting the background to minimize its impact, and then measuring the mean gray values. The expression levels of target proteins (TPH2, 5HTR1A, 5HTR2A) were normalized to the GAPDH levels within the same samples to account for any variations in protein loading. Normalized values were expressed as fold change relative to WT pre‐onset group. Three independent biological replicates (3 mice per group) were analyzed, and each sample was run in duplicate technical replicates.

Band saturation prevention: To ensure signals remained within the linear range, exposure times for each antibody were meticulously calibrated using a stepwise approach. For each blot, we performed preliminary 30‐s, 1‐min, 2‐min, and 5‐min exposures. The optimal exposure time (typically 1–2 min for TPH2, 2–3 min for 5‐HTR1A/5‐HTR2A, and 30–60 s for GAPDH) was selected as the highest exposure without any saturated pixels (pixel intensity < 255 in 8‐bit images). Saturation was checked using ImageJ's “Threshold” function with the “Saturation” indicator enabled.

Background subtraction: For densitometric analysis using ImageJ (version 1.8.0), background subtraction was performed using the “Rolling Ball” algorithm with a radius of 50 pixels. Alternatively, for each lane, a rectangle of identical size was drawn immediately adjacent to each band to measure local background, and the mean gray value of this background region was subtracted from the band measurement. All measurements were performed in triplicate for each band.

Normalization: The expression levels of target proteins (TPH2, 5‐HTR1A, 5‐HTR2A) were normalized to GAPDH levels from the same lane. Normalized values were expressed as fold change relative to WT pre‐onset group. Three independent biological replicates (3 mice per group) were analyzed, and each sample was run in duplicate technical replicates.

### 2.5 Randomization

In each genotype and disease stage group, mice were randomly allocated to tissue collection for histological or biochemical analysis using a random number table to ensure equal distribution across groups. For the histological assessment, sections from all groups underwent a standardized staining process and were subsequently analyzed in a randomized order to ensure unbiased evaluation.

### 2.6 Blinding

In the assessment of outcomes, the investigators were kept unaware of WT and TG mice and the progression of the disease in the samples to prevent bias. This included the histological scoring of muscle atrophy, the acquisition of microscope images, the quantification of immunofluorescent cells, and the analysis of western blot bands.

### 2.7 Data Analysis

The experimental data were presented as mean ± standard deviation. The statistical analysis of differences between two groups was conducted using a paired sample *t*‐test to account for the relatedness of the data. To assess the differences among multiple groups, a one‐way ANOVA was conducted followed by a Tukey post hoc test to identify specific group differences. Given the numerous group comparisons in this study (3 time points × multiple anatomical regions × 2 genotypes), the Benjamini–Hochberg To mitigate Type I errors, the False Discovery Rate (FDR) correction was implemented, setting the threshold at *q* < 0.05. Consequently, adjusted *p*‐values, also known as q‐values, were computed for all primary comparisons. Comparisons were deemed statistically significant only if the q‐value was less than 0.05 following the FDR correction, aligning with the conventional threshold for statistical significance. Spearman's rank correlation analysis was employed to assess the association between the quantity of 5‐HT‐positive cells and neuronal cells, with a *p*‐value adjustment < 0.05 signifying a statistically significant correlation.

## 3 Results

### 3.1 The Alteration of 5‐HT Distributed Features Among the Cervical, Thoracic, and Lumbar Segments of Adult Spinal Cord at the Pre‐Onset, Onset and Progression Stage Between WT and TG Mice

**FIGURE 1:**
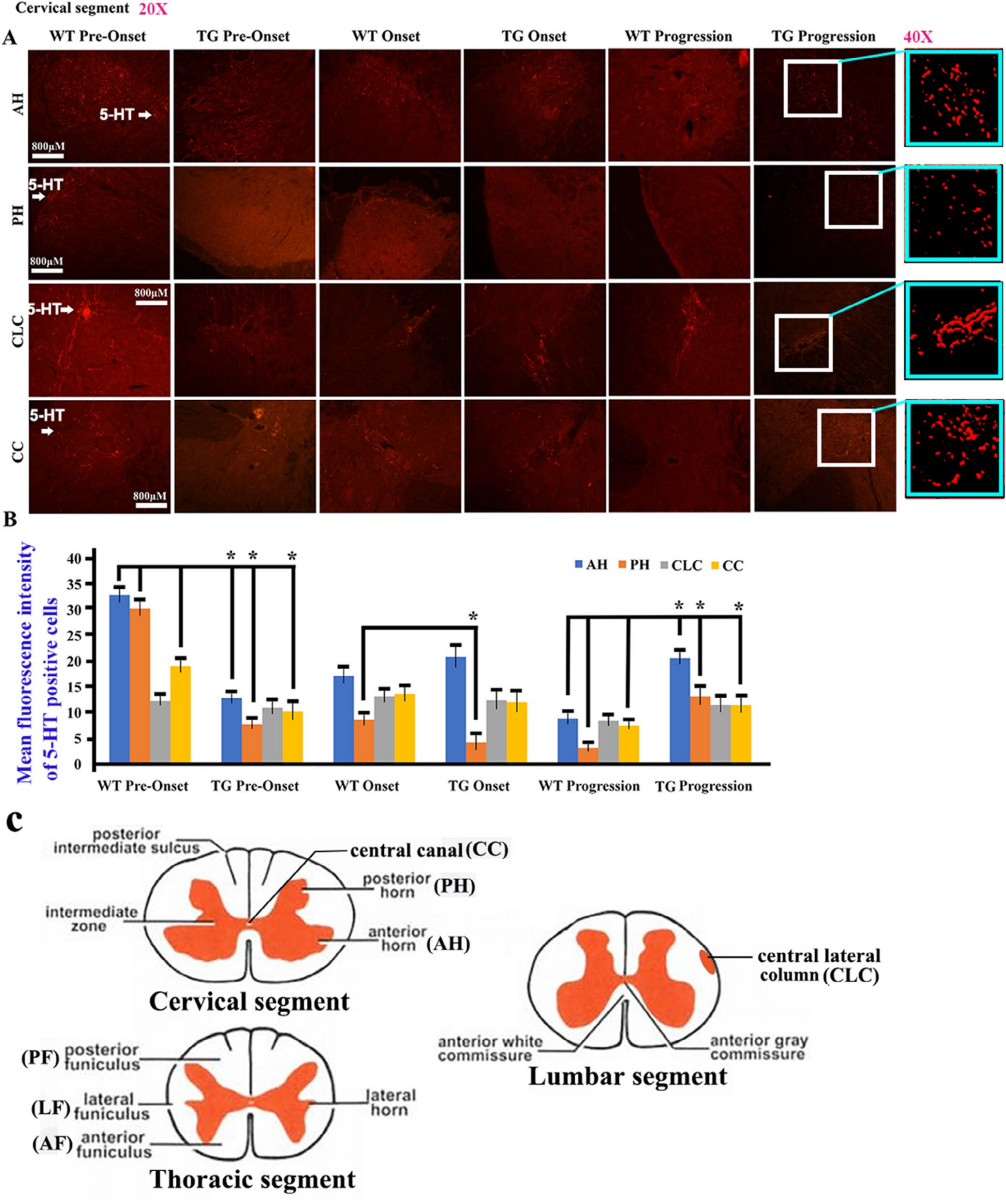
The 5‐HT distribution in the cervical AH, PH, CLC, and CC of adult spinal cord. (A) The representative images of 5‐HT distribution in the cervical segment of both WT and TG mice at the pre‐onset, onset, and progression stages. (B) The comparison of 5‐HT distribution in the adult cervical AH, PH, CLC, and CC between WT and TG mice at the pre‐onset, onset, and progression stages. Five mice per group. *p < 0.05. The 5‐HT distribution in the cervical AH, PH, and CC at the pre‐onset stage as well as in the cervical PH at the onset stage significantly decreased, but significantly increased in the cervical AH, PH, and CC at the progression stage in TG mice. (C) The schematic diagram of spinal cord anatomic regions. The schematics of the spinal cord in Figure 1C were downloaded from the free public websites (https://image.baidu.com/).

**FIGURE 2:**
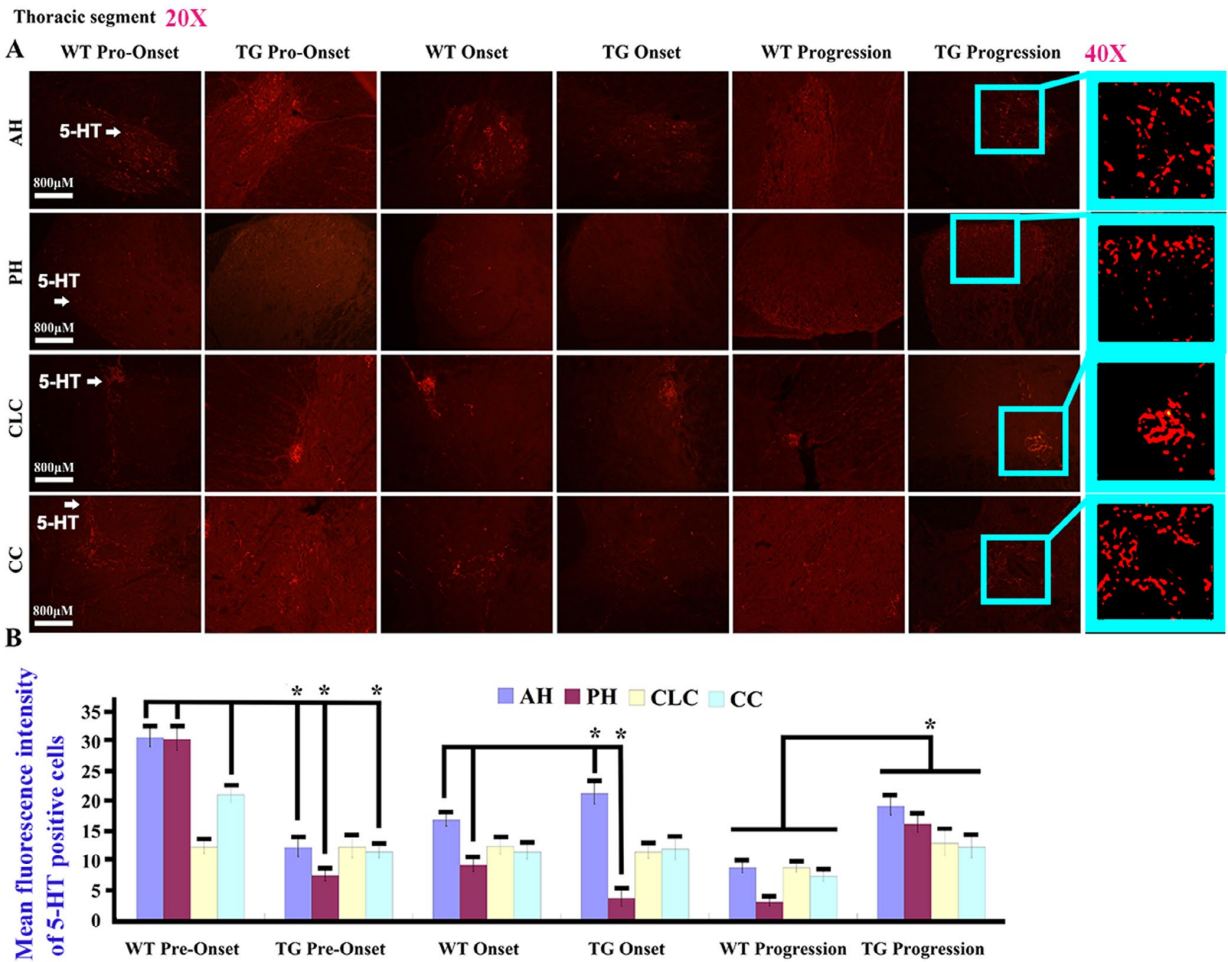
The 5‐HT distribution in the thoracic AH, PH, CLC, and CC of adult spinal cord. (A) The representative images of 5‐HT distribution in the thoracic segment of both WT and TG mice at the pre‐onset, onset, and progression stages. (B) The comparison of 5‐HT distribution in the adult thoracic AH, PH, CLC, and CC between WT and TG mice at the pre‐onset, onset, and progression stages. Five mice per group, *p < 0.05. The 5‐HT distribution in the thoracic AH, PH, and CC at the pre‐onset stages as well as in the thoracic AH and PH at the onset stage significantly decreased, but significantly increased in the thoracic AH, PH, CLC, and CC at the progression stage in TG mice.

**FIGURE 3:**
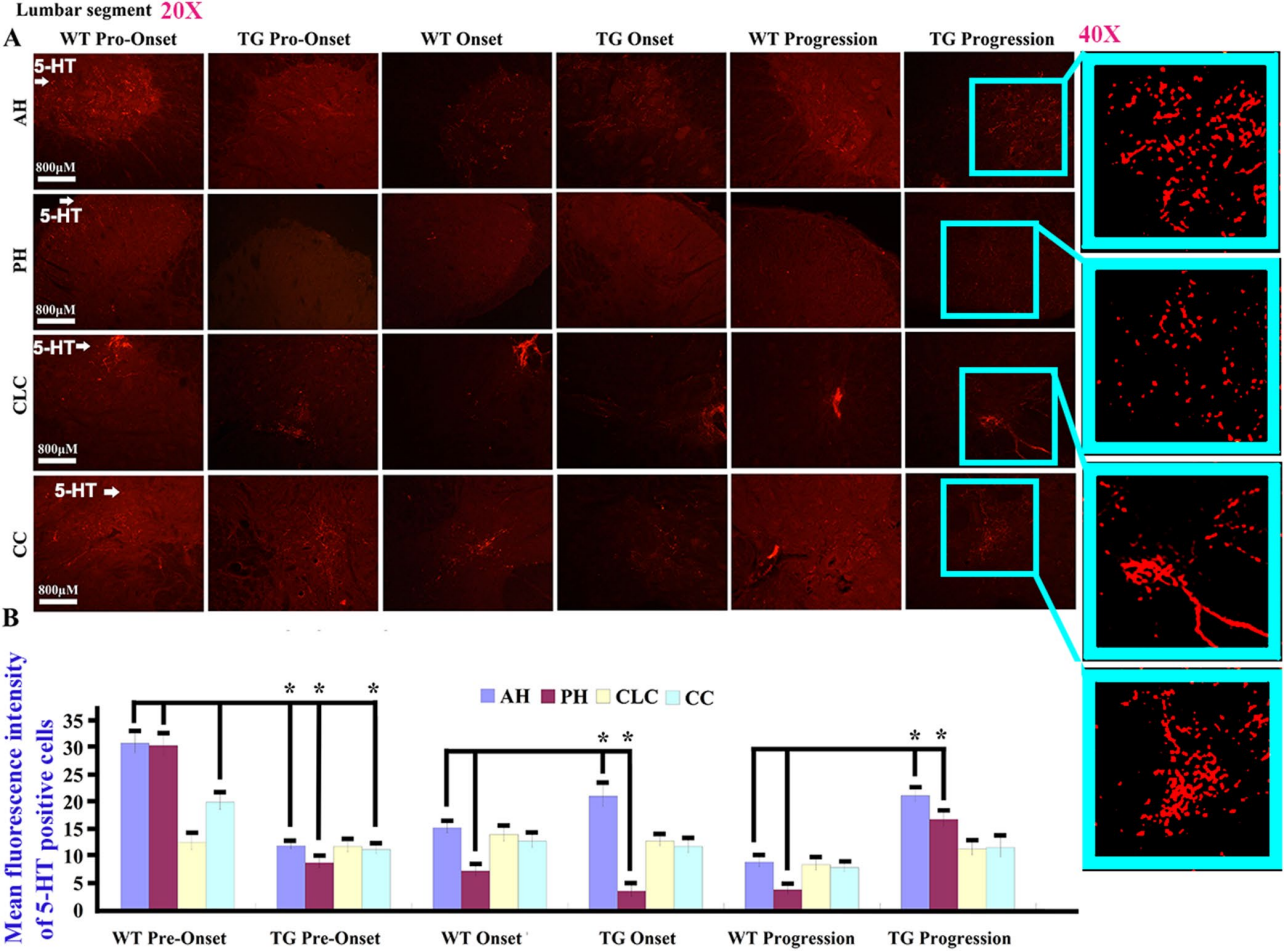
The 5‐HT distribution in the lumbar AH, PH, CLC, and CC of the adult spinal cord. (A) The representative images of 5‐HT distribution in the lumbar segment of both WT and TG mice at the pre‐onset, onset, and progression stages. (B) The comparison of 5‐HT distribution in the adult lumbar AH, PH, CLC, and CC between WT and TG mice at the pre‐onset, onset, and progression stages. In the experiment, five mice per group were used, and the results showed a statistically significant difference with a *p*‐value less than 0.05. The 5‐HT distribution in the lumbar AH, PH, and CC at the pre‐onset stage as well as in the lumbar PH at the onset stage significantly decreased, but significantly increased in the lumbar AH and PH at the progression stage in TG mice.

5‐HT mainly expressed in the gray matter of adult spinal cord, including the cervical, thoracic, and lumbar anterior horn (AH), posterior horn (PH), central lateral column (CLC), and around central canal (CC). In the spinal cervical segment, the 5‐HT distribution in AH, PH, and CC at the pre‐onset stage as well as that in PH at the onset stage showed a significant decrease, but that in AH, PH, and CC at the progression stage significantly increased while compared WT with TG mice (Figure 1A,B). In the thoracic segment, the distribution of 5‐HT in AH, PH, and CC at the pre‐onset stage as well as that in PH at the onset stage significantly decreased. However, when comparing WT with TG mice, that in AH at the onset stage and that in AH, PH, and CC at the progression stage significantly increased (Figure 2A,B). In the lumbar segment, the distribution of 5‐HT in AH, PH, and CC at the pre‐onset stage as well as that in PH at the onset stage exhibited a significant decrease, but that in AH at the onset stage and that in AH and PH at the progression stage significantly increased while compared WT with TG mice (Figure 3A,B).

**FIGURE 4:**
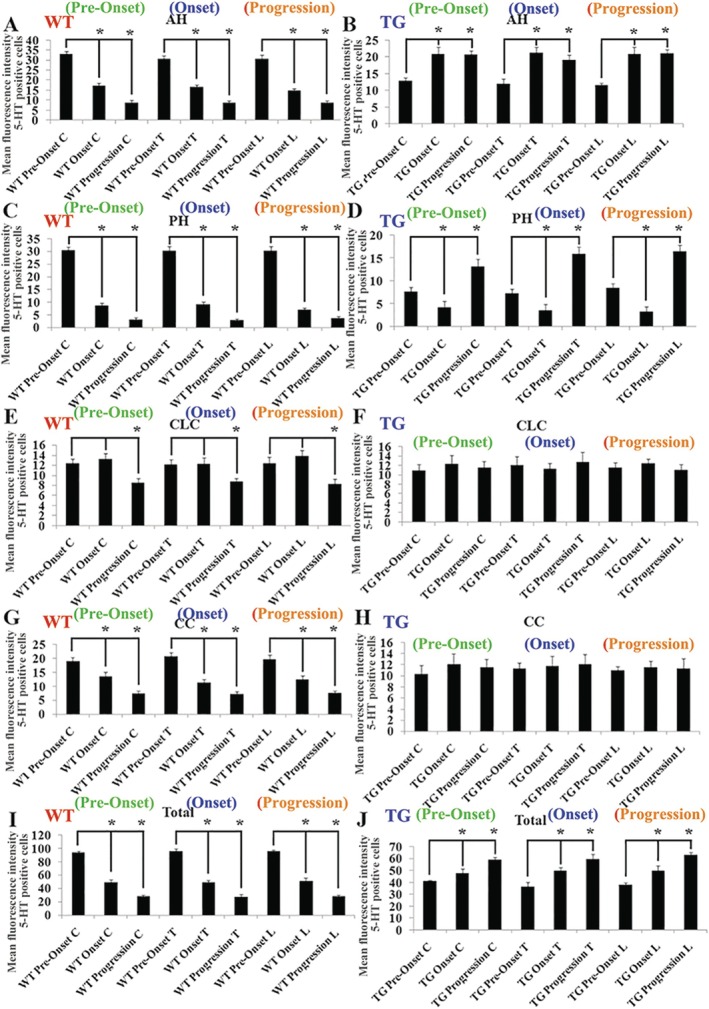
The comparison of 5‐HT distribution in AH, PH, CLC, CC, and total 5‐HT distribution in spinal cord between WT and TG mice. (A, B) The comparison of the 5‐HT distribution in the AH of different spinal cord segments of WT (A) and TG (B) mice at the pre‐onset, onset, and progression stages. (C, D) The comparison of the 5‐HT distribution in the PH of different spinal cord segments of WT (C) and TG (D) mice at the pre‐onset, onset, and progression stages. (E, F) The comparison of the 5‐HT distribution in the CLC of different spinal cord segments of WT (E) and TG (F) mice at the pre‐onset, onset, and progression stages. (G, H) The comparison of the 5‐HT distribution in the CC of different spinal cord segments of WT (G) and TG (H) mice at the pre‐onset, onset, and progression stages. (I, J) The comparison of total 5‐HT distribution in the different spinal cord segments of WT (I) and TG (J) mice at the pre‐onset, onset, and progression stages. In an experiment involving five mice per group, a statistically significant result was observed with a *p*‐value less than 0.05.

The comparison of 5‑HT distribution in the cervical, thoracic, and lumbar AH, PH, CC, and CLC, as well as the entire spinal cord, during the pre‑onset, onset, and progression stages (Figure 4) revealed that, in wild‑type mice, 5‑HT levels in these regions were significantly reduced at the onset and progression stages of the condition (Figure 4A–I). Additionally, the distribution of 5‐HT in the cervical, thoracic, and lumbar CLC of these mice at the onset and progression stages (Figure 4E) showed a marked decrease compared to the pre‐onset stage, as reported in previous studies. Meanwhile, the 5‐HT distribution in the AH and their entire cervical, thoracic, and lumbar segments of TG mice at the stage of onset and progression (Figure 4B–J) as well as that in PH at the progression stage (Figure 4D) showed a significant increase, and the 5‐HT distribution in the cervical, thoracic, and lumbar PH of TG mouse at the onset stage significantly decreased compared with the pre‐onset stage. The comparison of 5‐HT distribution in the cervical, thoracic, and lumbar CLC and CC of TG mice at the pre‐onset, onset, and progression stages did not reveal any significant changes (Figure 4F–H).

**FIGURE 5:**
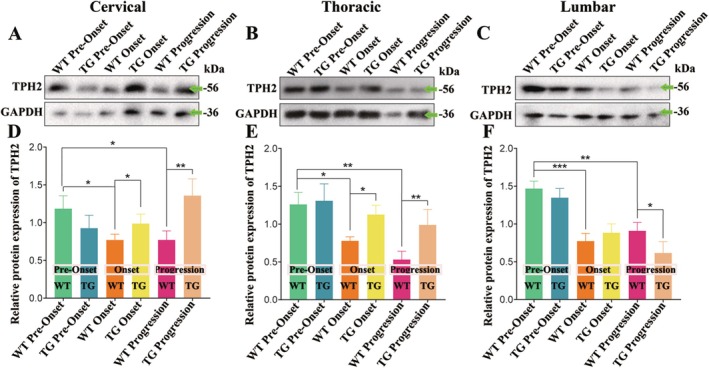
The alterations of TPH2 level in the adult spinal cords at the different stages of TG mice and the age‐matched same period of WT mice. (A–C) The representative western blot bands of TPH2 in the cervical, thoracic, and lumbar segments of spinal cord, respectively. (D–F) Western blot analysis, a widely used technique for protein detection and quantification, was employed to assess the expression levels of TPH2 in the cervical, thoracic, and lumbar segments of the spinal cord at pre‐onset, onset, and progression stages of TG mice, as well as in age‐matched WT mice. Nine mice per group. Target protein TPH2, expected molecular weight~56 kDa, antibody source (Catalog# ab211528, Abcam). Target protein GAPDH, expected molecular weight~36 kDa, antibody source (Catalog# ab245355, Abcam).

To further confirm the alterations of 5‐HT expression in spinal cord with the disease progression, western blot analysis was used to examine the TPH2 level in cervical, thoracic, and lumbar segments of spinal cord at the different stages of TG mice and the age‐matched same period of WT mice. In the cervical segments, TPH2 expression was decreased at the onset stage and progression stage of WT mice, while increased at the progression stage of TG mice (Figure 5A,D). In the thoracic segments, TPH2 expression gradually decreased at onset stage and progression stage of WT mice, while significantly increased at the onset stage of TG mice (Figure 5B,E). In the lumbar segments, TPH2 expression gradually decreased at onset stage and progression stage of WT mice (Figure 5C,F). These results confirmed that the TPH2 expression in the entire spinal cord of WT mice showed a significant decrease at the onset and progression stages, while the cervical segments of spinal cord in TG mice at the progression stage and the thoracic segments of the spinal cord in TG mice at the onset stage showed a significant upregulation of TPH2.

**FIGURE 6:**
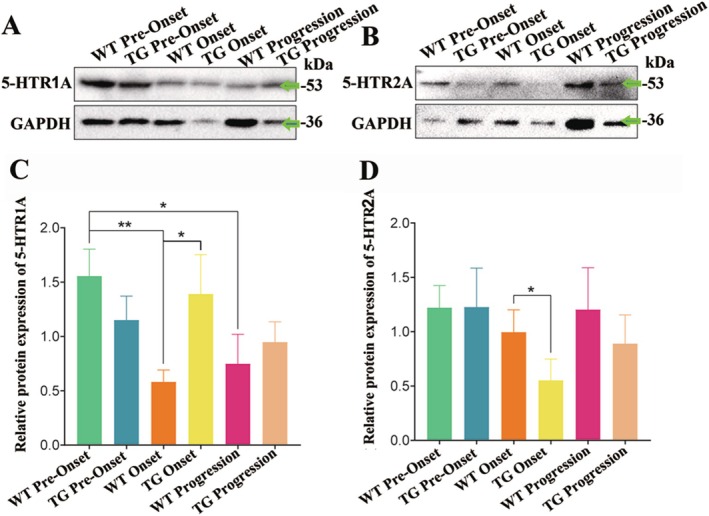
The alterations of both 5‐HTR1A and 5‐HTR2A levels in the adult spinal cords at the different stages of TG mice and the age‐matched same period of WT mice. (A, B) The representative western blot bands of 5‐HTR1A and 5‐HTR2A in the thoracic spinal cord, respectively. (C, D) Western blot analysis showing the protein levels of both 5‐HTR1A and 5‐HTR2A in the thoracic spinal cord at the pre‐onset, onset, and progression stages of TG mice and the age‐matched same period of WT mice. Nine mice per group. Target protein 5‐HTR1A and 5‐HTR2A, expected molecular weight~46 kDa and~53 kDa, antibody source (Catalog# AF5453, Affinity Biosciences) and (Catalog# bs‐12049R, Biosynthesis). Target protein GAPDH, expected molecular weight~36 kDa, antibody source (Catalog# ab245355, Abcam).

Next, we further investigated whether specific 5‐HT receptor subtypes were increased in ALS. 5‐HT_1A_R (also known as 5‐HTR1A), which is a highly characterized spinal 5‐HTR, is reported to increase spinal MN excitability [36]. The expression of 5‐HTR1A in the thoracic segments of the spinal cord was evaluated at three stages in both WT and TG mice. Western blot analysis revealed that the level of 5‐HTR1A significantly decreased at both onset and progression stages compared with the pre‐onset stage in WT mice, while there were not significant changes from pre‐onset to onset to progression stage in TG mice. However, the level of 5‐HTR1A in TG mice significantly increased at the onset stage compared with WT mice (Figure 6A,C). 5‐HTR2A is another 5‐HT receptor subtype which is reported to be involved in mediating 5‐HT effects on MN [37]. However, 5‐HTR2A expression remained similar across all three stages in WT mice, whereas it significantly decreased at the onset stage in TG mice (Figure 6B,D). These results suggested that 5‐HTR1A, but not 5‐HTR2A, might be involved in ALS. However, the involvement of other 5‐HT receptor subtypes in ALS cannot be ruled out yet.

### 3.2 The Alteration of TPH2 Distributed Features in the Cervical, Thoracic, and Lumbar Segments of Adult Spinal Cord at the Pre‐Onset, Onset, and Progression Stage Between WT and TG Mice

**FIGURE 7:**
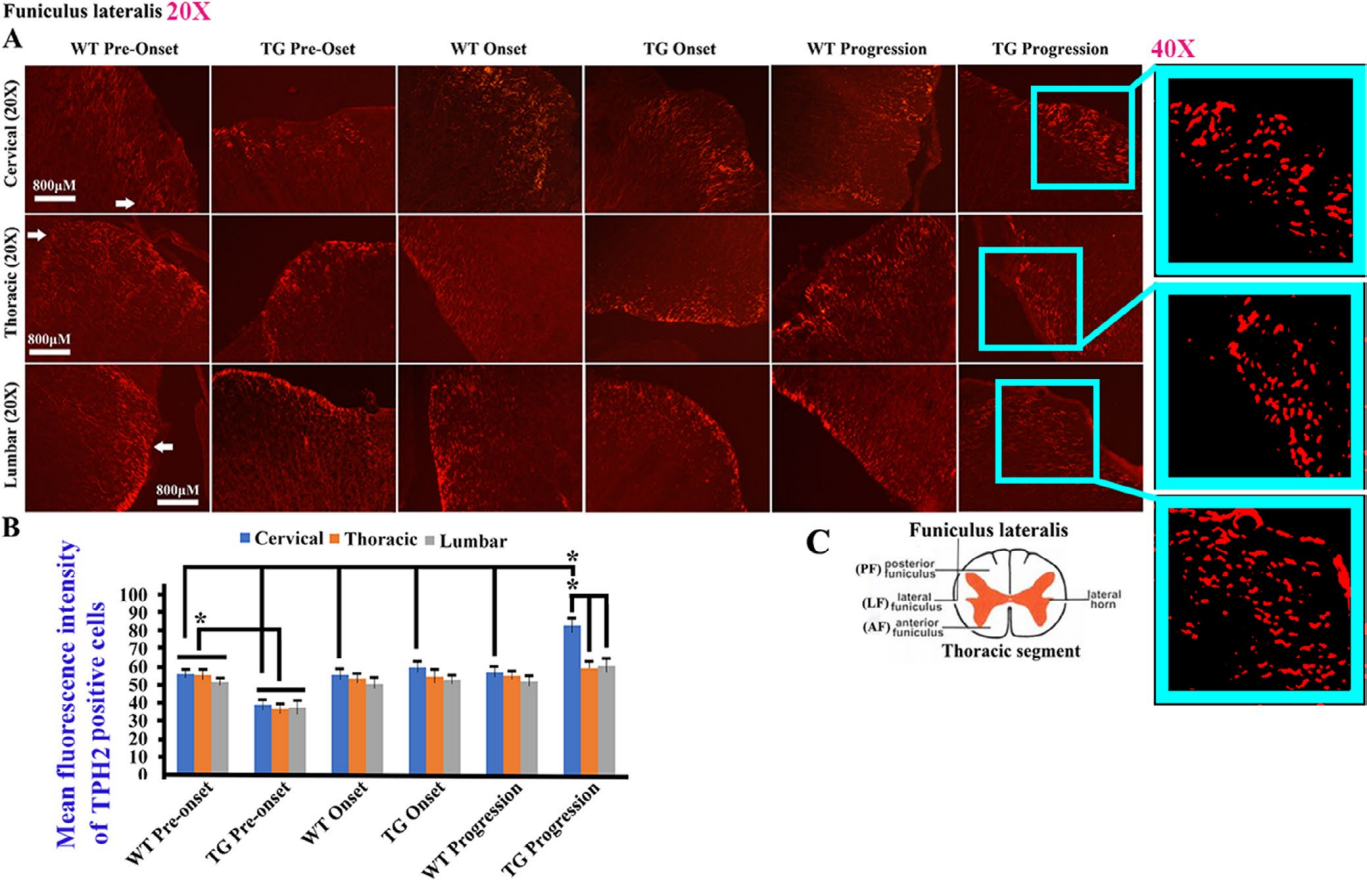
The TPH2 distribution in the funiculus lateralis (FL) of adult spinal cord. (A) The representative images of TPH2 distribution in the cervical, thoracic, and lumbar FL at the pre‐onset, onset, and progression stages of both WT and TG mice. Five mice per group; *p < 0.05. FL exhibited the highest TPH2 distribution in the adult spinal cord. (B) The comparison of TPH2 distribution in the cervical, thoracic, and lumbar FL at the pre‐onset, onset, and progression stages of both WT and TG mice. Five mice per group, *p < 0.05. The TPH2 distribution in the cervical, thoracic, and lumbar FL significantly decreased at the pre‐onset stage, but that in the cervical segment significantly increased at the progression stage in TG mice. (C) The schematic diagram of spinal cord anatomic regions. The schematic of the spinal cord in Figure 7C was downloaded from the free public websites (https://image.baidu.com/).

TPH2 is mainly expressed in the adult spinal white matter, particularly in the funiculus lateralis (FL) (Figure 7A). When comparing WT and TG mice, TPH2 expression in the FL is significantly reduced at the pre‐onset stage, whereas in the cervical segment, TPH2 expression shows a significant increase at the progression stage (Figure 7B). The TPH2 at the progression stage, the distribution in the cervical segment was greater than that in both the thoracic and lumbar segments, TPH2 did not show a significant difference between the cervical, thoracic, and lumbar segments at both the pre‐onset and onset stages (Figure 7B). In the cervical segment, the TPH2 distribution at the progression stage was significantly higher than that at both the pre‐onset and onset stages, representing the highest distribution (Figure 7B).

### 3.3 The Overlapped Distribution TPH2, 5‐HT, DAPI, GFAP, Vimentin, and NeuN in the Adult Spinal Cord

**FIGURE 8:**
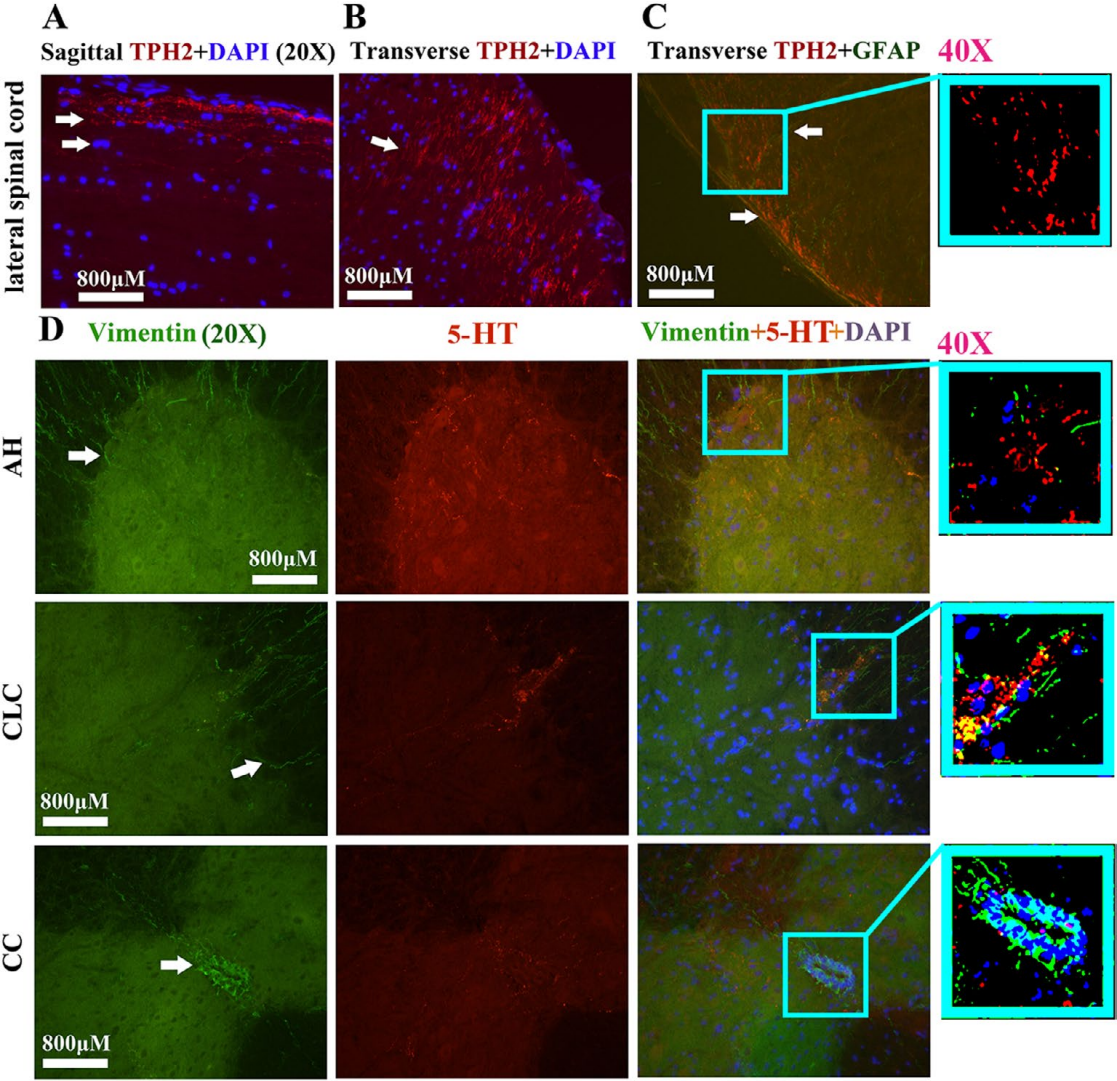
The double staining results for TPH2/DAPI/GFAP and 5‐HT/Vimentin. (A, B) Representative images showing the double staining of TPH2 and DAPI in the sagittal (A) and transverse (B) sections of the spinal cord. (C) Representative images showing double staining of TPH2 and GFAP in the transverse section of the spinal cord. (D) The representative images of 5‐HT and Vimentin double stain in the spinal AH, CLC, and CC. The distribution of TPH2 was not doubly stained with DAPI, GFAP, and Vimentin. The scale bar of all immunofluorescence images in Figure 8 was 800 μm.

**FIGURE 9:**
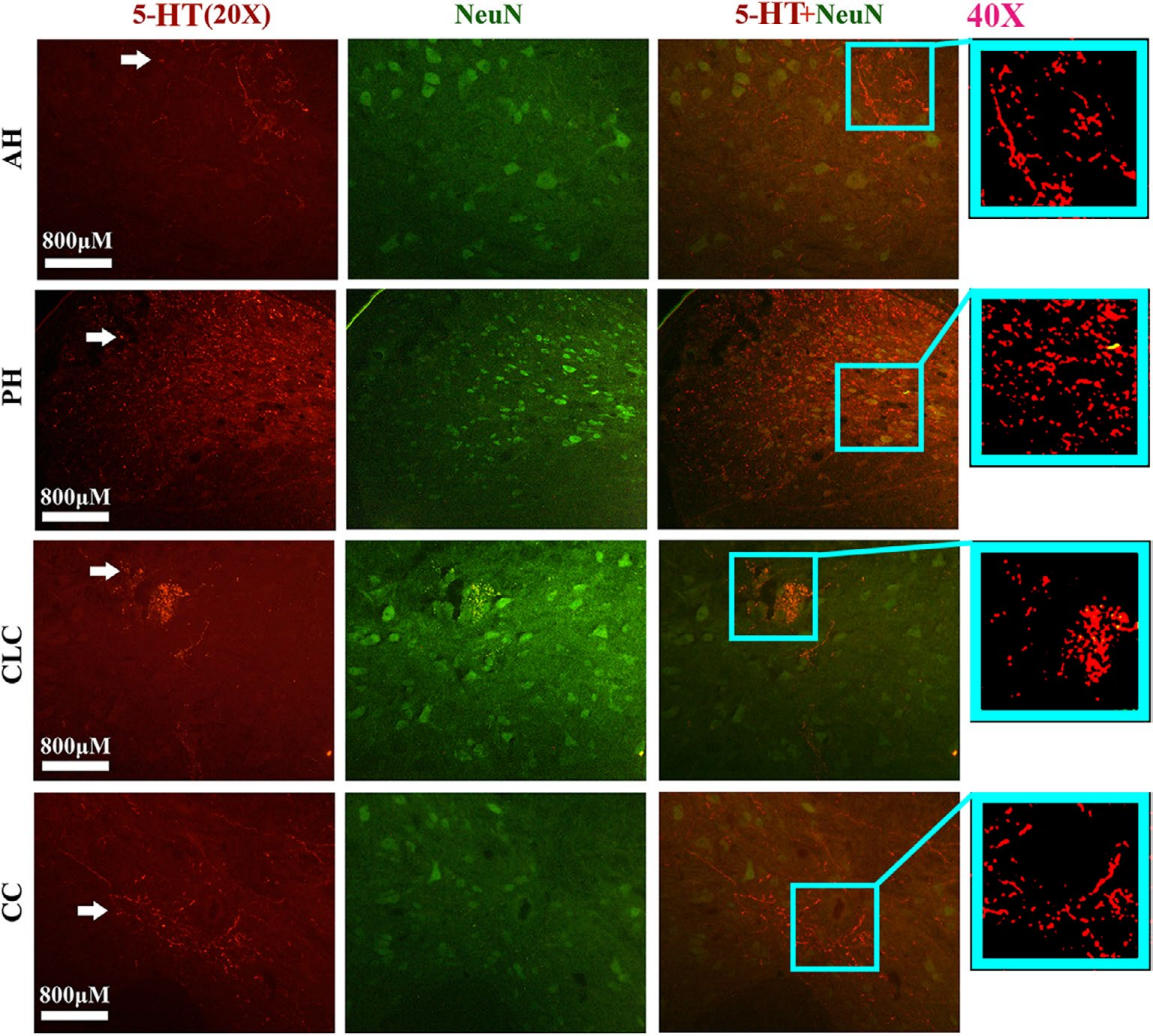
The double stain of 5‐HT and NeuN in the AH, PH, CLC, and CC of adult spinal cord. The distribution of 5‐HT was not doubly stained with NeuN in the spinal AH, PH, CLC, and CC. The scale bar of all immunofluorescence images in Figure 9 was 800 μm.

The representative images show overlapped staining of TPH2 and DAPI in the lateral sagittal and transverse spinal cord (Figure 8A,B). The representative images show overlapped staining of TPH2 and GFAP in the lateral transverse spinal cord (Figure 8C). The representative images show overlapped staining of 5‐HT and Vimentin (Figure 8D) and 5‐HT and NeuN (Figure 9) in the AH, PH, CLC, and CC of lateral transverse spinal cord; no overlapped staining was observed among them. The results indirectly indicated that TPH2 and 5‐HT were not expressed in neuronal bodies, astrocytes, neural precursor cells (NPCs), or neurons. Furthermore, based on the anatomical distribution features of neuronal synapses in the adult spinal cord, the results indirectly suggested that TPH2 and 5‐HT were expressed in neuronal synapses.

### 3.4 The Alteration of TPH2 Distributed Features in the Adult Brainstem at the Pre‐Onset, Onset, and Progression Stage Between WT and TG Mice

**FIGURE 10:**
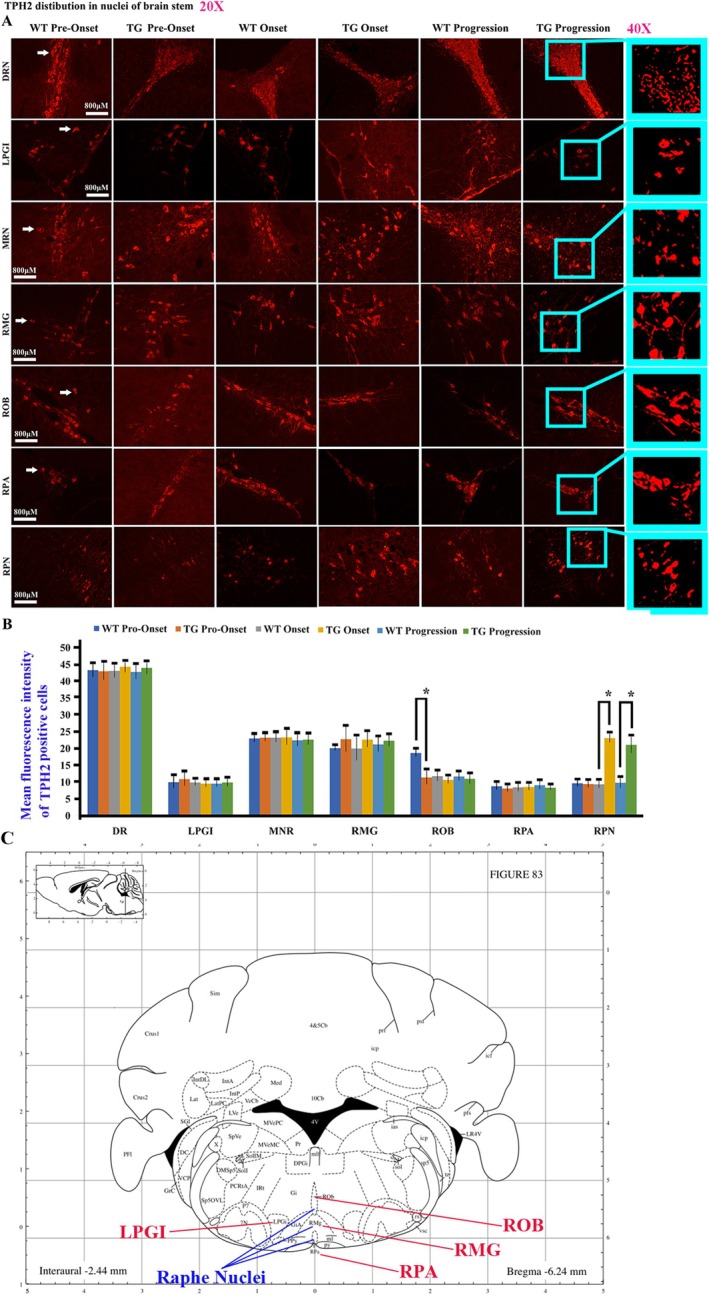
The TPH2 distribution in the nuclei of adult brainstem. (A) The representative images of TPH2 distribution in the different regions of adult brainstem of both WT and TG mice at the pre‐onset, onset, and progression stages. (B) The comparison of TPH2 distribution in the different nuclei of adult brainstem between both WT and TG mice at the pre‐onset, onset, and progression stages. In the experimental setup, five mice were used per group, and the results showed statistical significance with a *p*‐value less than 0.05. The TPH2 distribution in the ROB nucleus of adult brainstem significantly decreased at the pre‐onset stage, but that in RPN significantly increased at both onset and progression stages in TG mice. (C) The schematic diagram of spinal cord anatomical regions. The schematics of brain stem in Figure 10C were cited from the second edition of mouse brain stereotaxic coordinates edited by professor George Paxinos and professor Keith B. J. Franklin.

TPH2 mainly expressed in the nuclei of the brainstem, including the dorsal raphe nucleus (DRN), median raphe nucleus (MRN), raphe magnus nucleus (RMG), raphe obscurus nucleus (ROB), raphe pallidus nucleus (RPA), raphe pontine nucleus (RPN), and lateral paragigantocellular nucleus (LPGI). In WT mice, the TPH2 distribution was most abundant in the DRN, followed by the MNR and RMG, and then the ROB, RPA, RPN, and LPGI. While compared between WT and TG mice, TPH2 in the ROB at the pre‐onset stage significantly decreased, TPH2 in RPN at both onset and progression stages significantly increased (Figure 10B).

### 3.5 The Overlapped Distribution of TPH2, Nestin, NeuN, and Vimentin in the Adult Brainstem

**FIGURE 11:**
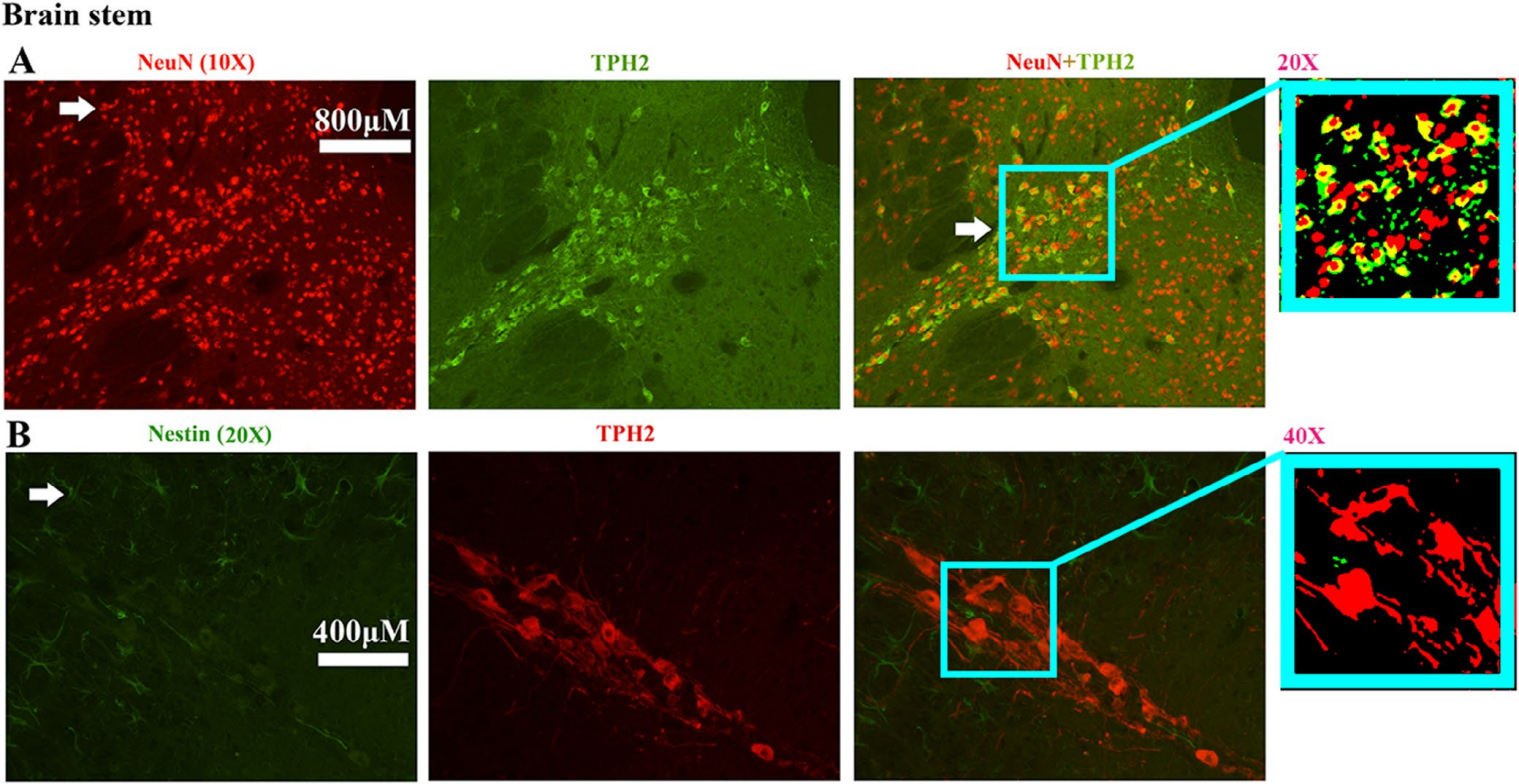
The double staining of TPH2/NeuN/Nestin in the adult brainstem. (A) Representative images showing the double staining of TPH2 and NeuN in the adult brainstem. The distribution of TPH2 that is doubly stained with NeuN in the adult brainstem. (B) Representative images showing double staining of TPH2 and Nestin in the adult brainstem. TPH2 was not doubly stained with Nestin in the brainstem.

The representative images of double staining of TPH2 with Nestin, NeuN, and Vimentin showed that all TPH2 overlapped with NeuN (Figure 11A), did not detect any overlapped staining with Nestin (Figure 11B) or Vimentin (Figure 11C). The results showed that TPH2 was expressed in neurons, but not in NPCs in the brainstem.

### 3.6 The Correlation Between Neural Cell Number and Both 5‐HT and TPH2 Distribution in the Spinal Cord and the TPH2 Distribution in the Brainstem

**FIGURE 12:**
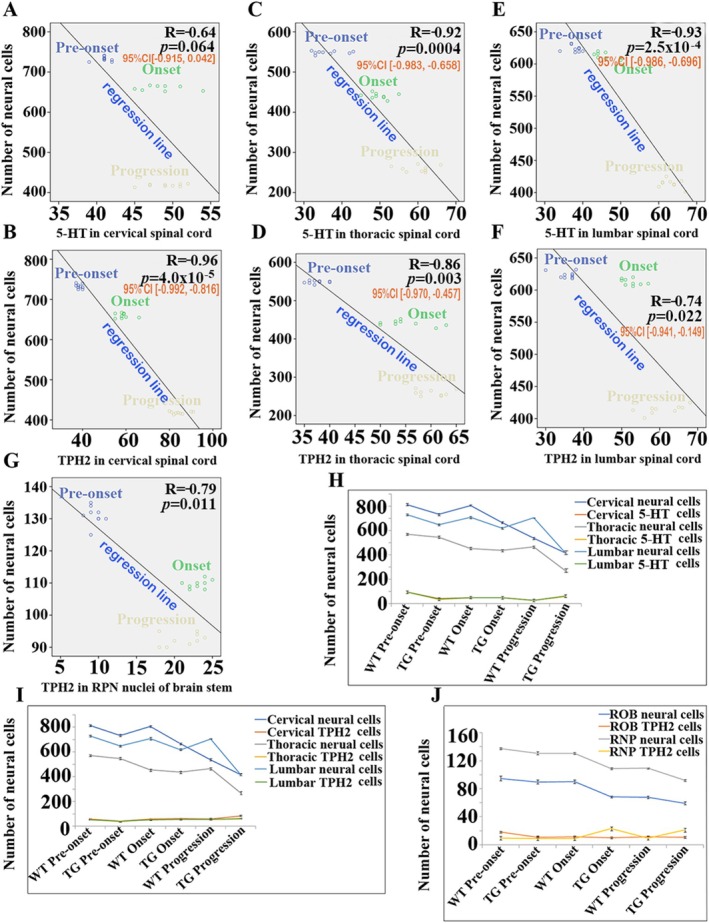
The correlation between the distribution of 5‐HT and TPH2 and neural cell death in both spinal cord and brainstem at the pre‐onset, onset, and progression stages of TG mice. (A–F) The correlation between the distribution of 5‐HT (A, C, E) and TPH2 (B, D, F) and the number of neural cells (a decrease in neural cell number indirectly implies an increase in neural cell death) in the cervical, thoracic, and lumbar segments across the pre‐onset, onset, and progression stages. Nine mice per group. (G) The correlation between the TPH2 distribution and the number of neural cells in the RPN nuclei of brainstem at the pre‐onset, onset, and progression stages. Nine mice per group. The 5‐HT distribution in both thoracic and lumbar segments, the TPH2 distribution in the spinal cervical, thoracic, and lumbar segments as well as TPH2‐positive cells in the RPN nuclei of brainstem presented a significantly negative correlation with the number of neural cells at the onset and/or progression stage of TG mice, which implied that studies have shown that, during the onset and progression of pathological conditions, there is an increase in the levels of 5‐HT and TPH2, which are associated with neuronal death. (H–J) The alteration tendency between 5‐HT (H) and TPH2 (I) distribution in spinal cord and TPH2 positive cells (J) in brainstem as well as the number of neural cells at the pre‐onset, onset, and progression stages of both WT and TG mice. The increase of both 5‐HT and TPH2 distribution was accompanied with the decrease in number of neural cells (the neural cell death increase) in the spinal cervical, thoracic, and lumbar segments in TG mouse. The increase of TPH2 distribution was accompanied by the neural cell death increase in the RPN nuclei of brainstem in TG mouse. Five mice per group.

The reduction of neural cell number showed a negative correlation with 5‐HT and TPH2 distribution increase in the spinal cervical (Figure 12A) (correlation coefficients (*r* values) (R) = −0.64, 95% confidence interval (CI) [−0.915, 0.042], *p* = 0.064) and B (R = −0.96, 95% CI [−0.992, −0.816], *p* = 4.0 × 10^−5^), thoracic (Figure 12C (R = −0.92, 95% CI [−0.983, −0.658], *p* = 0.0004) and D (R = −0.86, 95% CI [−0.970, −0.457]), *p* = 0.003) and lumbar (Figure 12E (R = −0.93, 95% CI [−0.986, −0.696], *p* = 2.5 × 10^−4^) and F (R = −0.74, 95% CI [−0.941, −0.149]), *p* = 0.022) segments and the TPH2 distribution increase in the RNP nuclei (Figure 12G (R = −0.79, 95% CI [−0.954, −0.265]), *p* = 0.011) of brainstem in TG mice, which indirectly implied that the alteration of 5‐HT and TPH2 distribution in both spinal cord and brainstem exhibited a positive correlation with neural cell death in both spinal cord and brainstem 5‐HT nuclei (Figure 12H–J). Results reminded us that the increase of both 5‐HT and TPH2 distribution in both spinal cord and brainstem might be one of potential candidate factors in neurons' death in the ALS pathogenesis.

## 4 Discussion

In our study, we observed and analyzed the distributed features of 5‐HT neurotransmitter in the different anatomical regions and segments of spinal cord and the different brainstem nuclei in both WT and TG mice applying fluorescence immunohistochemistry staining of the 5‐HT and TPH2 biomarkers; compared their distribution at the pre‐onset, onset, and progression stages between WT and TG mice; and observed the relationship between 5‐HT distribution and neurons, glial cells, and NPCs. Our results showed that the 5‐HT biomarker mainly distributed in the spinal AH, PH, CLC, and CC of gray matter with the stripe shape (Figures 1, 2, 3), the distribution in both AH and PH was the most abundant, and the secondary was in the cervical, thoracic, and lumbar CLC and CC (Figures 1, 2, 3). The 5‐HT distribution in the AH, PH, CLC, and/or CC significantly decreased during the pre‐onset stages (Figures 1, 2, 3, 4, 5, 6, 7) when comparing TG mice with WT mice; however, it significantly increased during the progression stages in TG mice. Although the mechanism underlying the abnormal 5‐HT distribution in the spinal cord of TG mice remained unclear in our study, it suggested that the abnormal alteration of 5‐HT distribution occurred in the AH, PH, and/or CC, especially in the AH. The abnormal distribution of 5‐HT in the spinal cord of TG mice was in agreement with the damaged regions of spinal cord in ALS, such as AH. Therefore, recent studies suggest that the aberrant distribution of 5‐HT in the spinal cord of TG mice, particularly those expressing the G93A‐SOD1 mutation, may contribute to the pathogenesis of ALS by being associated with neuronal death.

TPH2 biomarker mainly distributed in the FL of spinal white matter by the linear shape, in the pre‐onset stage of ALS, a significant decrease in the distribution of the cervical, thoracic, and lumbar FL was observed, with a notable increase in the cervical segment during the progression stage (Figure 7). These distribution changes in 5‐HT are believed to be responsible for the spinal FL damage in the majorly affected regions of ALS. Consequently, we hypothesize that the abnormal distribution of 5‐HT in the spinal cord is closely linked to the FL damage in ALS. Both 5‐HT and TPH2 biomarker in spinal cord did not overlap stain with the neuron, glial, and NPC biomarker (Figures 7 and 8). We hypothesized that the stripe—or linear—distributed 5‐HT and TPH2 biomarkers in the spinal cord represented 5‐HT synapses projecting into the spinal cord, based on the anatomical features of 5‐HT synapse distribution, as the synapses of 5‐HT neurons comprehensively projected into the FL of the white matter and the AH, PH, CLC, and CC of the gray matter in the spinal cord [38, 39]. Meanwhile, we conducted the comparison of 5‐HT synapse in spinal cord between WT and TG mice; results showed the 5‐HT synapse in the spinal cervical, thoracic, and lumbar AH, PH, CC, and/or FL significantly reduced at both pre‐onset and/or onset stages; however, 5‐HT synapse in the cervical, thoracic, and lumbar AH, PH, CLC, CC, and/or FL significantly increased at the progression and/or stages (Figures 1, 2, 3, 4, 5, 6, 7). Our data revealed that the 5‐HT synapse in AH reduced at the pre‐onset stage, but that at the stage of both onset and progression increased in comparison of WT mice, which further identified that the abnormal distribution of 5‐HT was closely associated with neuronal damage in the AH during the pathogenesis of ALS.

In the brainstem, 5‐HT neurons are predominantly located within DRN, MNR, and RMG, with secondary abundance in ROB, RPA, RPN, and LPGI. The DRN exhibits the highest concentration of 5‐HT neurons, followed by the MNR and RMG, with a lesser but significant presence in the ROB, RPA, RPN, and LPGI (Figure 10A,B). The TPH2 biomarker in brainstem only overlapped with the neuron biomarker, which indicated that the TPH2 biomarker expressed cells were the neuronal cells (Figure 11). Our results further identified that the 5‐HT neuron existed in the DRN, MNR, RMG, ROB, RPA, RPN, and LPGI nuclei of brainstem [39]. Our results showed that 5‐HT neurons in the ROB of brainstem significantly reduced at the pre‐onset stage; however, 5‐HT neurons in the RPN of brainstem demonstrated a significant increase at the stage of both TG onset and progression in TG mice. Therefore, our data further revealed that recent studies have shown that the abnormal alteration of 5‐HT neuron distribution is closely associated with neural cell death in the brainstem during the progression of ALS, particularly within the RPN nuclei.

TPHs are enzymes that synthesize the 5‐HT neurotransmitter. Tyrosine, phenylalanine, and tryptophan hydroxylases constitute the biopterin‐dependent aromatic amino‐acid hydroxylase family. It employs one additional cofactor, iron. Human and other mammal animals have two unique TPH genes. Human TPH gene distributes in the 11th and 12th chromosome and encodes two distinct homogenous enzymes, such as TPH1 and TPH2 [40]. TPH1 is predominantly expressed in peripheral tissues that synthesize the 5‐HT neurotransmitter, such as the skin, gut, and pineal gland. Additionally, TPH1 is also expressed in the CNS. TPH2, which is specifically expressed in neurons and is a dominant isotype in the CNS, acts as a key rate‐limiting enzyme in the synthesis of 5‐HT. It catalyzes the addition of a hydroxyl group to the fifth position of tryptophan to produce 5‐HT amino acids, an initial and rate‐limiting step in 5‐HT production synthesizing 5‐HT neurotransmitter. TPH2 is an enzyme that synthesizes melatonin. TPH2 is a homologous enzyme of TPH in vertebrate animals. Human TPH2 mainly expresses among the brain 5‐HT neuron and largely distributes among midbrain RN [41, 42]. Therefore, we labeled 5‐HT neurons in both spinal cord and brainstem applying TPH2 fluorescent staining. Our results revealed that 5‐HT synapses comprehensively projected into the FL, AH, PH, CLC, and CC as well as both cortex and subcortex, and 5‐HT neurons mainly distributed in the RN nuclei of brainstem including DRN, MNR, RMG, ROB, RPA, RPN, and LPGI nuclei. Moreover, the abnormal alteration in the 5‐HT neuron distribution of both ROB and RPN nuclei was found at the onset and/or progression stages of TG mice; a significant decrease in the ROB nuclei at the onset stage and a significant increase in the RPN at both onset and progression stages were showed. Our data further indicated that 5‐HT neurotransmitter plays a role in the pathogenesis of G93A‐SOD1 transgenic mice, as suggested by studies examining the impact of androgens on the disease model because the 5‐HT neurons in ROB extensively project into both spinal cord and medulla nuclei including the ambiguous nucleus [38], and that in RPN mainly project into the various constructs of cortex and sub‐cortex [39], and might exist a potential relationship to the neuronal damage in the commonest damaged region in ALS, such as cortex and subcortex, spinal cord, and brainstem nuclei, especially in the MN of AH, cortex, and subcortex MN as well as the ambiguous nucleus of medulla.

RN is a moderate‐sized cluster of nuclei in the brainstem. Based on the sequence from caudal to rostral, the RN is divided into the ROB, RPA, RMG, RPN, MNR, DRN, and the caudal linear nucleus. Overall, all caudal RN, including RMG, RPA, and ROB, project to both the spinal cord and the brainstem [38]. The more rostral nuclei, including the RPN, MNR, and DRN, project toward brain areas of higher function [38]. In our study, we observed that the number increase of 5‐HT neurons in RPN presented a significantly positive correlation with the neural cell death. In TG mice, neural cell alterations were observed at both the onset and progression stages (Figure 12G,J). This indicates that the increased alteration of 5‐HT neurons projecting into brain areas of higher function may involve damage to motor and/or non‐MN in the cerebrum, which is one of the possible factors by which non‐MN are involved in sALS pathogenesis.

5‐HT nuclei are usually classified into two major clusters: the caudal and rostral groups, which contain three and four nuclei, respectively. The caudal group consists of ROB (B2 nucleus), RMG (also known as B3 nucleus), RPA (B1 nucleus), and the lateral medullary reticular nucleus (LMR), which project into the brainstem and spinal cord, including the FL, AH, PH, CLC, and CC as well as the brainstem‐related regions, which involved in the motor activity of both spinal cord and brainstem [38, 39]. Rostral cluster composes of caudal linear nucleus (CLN) (B8 nucleus), DRN (B6 and B7 nucleus), MNR (B5, B8, and B9 nucleus), and RPN, projects to various constructs of cortex and subcortex [39]. RMG, ROB, RPA, and LMR projecting into both brainstem and spinal cord, and the CLN, DRN, MNR, and RPN project into the cortical and subcortical regions is involved in motor functions through pathways of 5‐HT [38, 39]. Our results showed that the 5‐HT ROB projected into the spinal cord and brainstem, and RPN nuclei projected into various constructs of the cortex and subcortex, significantly increased following the sALS progression, respectively, which further indicated that the increase of both 5‐HT neuron and recent studies have shown that alterations in the 5‐HT system within the brainstem are closely linked to the pathogenesis of sALS, particularly concerning MN and non‐MN death in the spinal cord, brainstem as well as cortical and subcortical regions.

Our data indicate a significant association between the abnormal alterations of 5‐HT synapses in the spinal cord and 5‐HT neurons in the brainstem with the pathogenesis of ALS. However, the precise nature of these 5‐HT alterations in both regions remains to be elucidated, either damage or protect neural cells during the development of sALS. The 5‐HT level in platelets showed a significant decrease in sALS patients. 5‐HT levels in platelets were not correlated to sALS duration, while showed a positive correlation to the lifetime of sALS patient [43]. Plasma uncombined 5‐HT and 5‐hydroxyindoleacetic acid level in plasma did not change in sALS patients in comparison with control groups and was not related to clinical parameters [44]. The current research suggested that the changes of 5‐HT levels in CNS might be important parameters estimating the pathogenesis, prognosis, and therapy of sALS; the therapy moderating 5‐HT neurotransmitter is obvious to be one of the candidate treatment targets according to the pharmacologic perspectives [45]. By extension, the 5‐HTergic denervation is speculated to result in the loss of function inhibiting glutamate release, which could potentially lead to glutamate‐induced neuronal toxicity in the inferior and superior motor neurons, and the 5‐HT facilitating effect regulating the excitability of glutamatergic MN viciously decreases. Therefore, we conducted the hypothesis that 5‐HT neurotransmitter might play a potential neuronal protective role in sALS, probably via 5‐HTR1A. Although selective 5‐HT reuptake inhibitors such as some antidepressant drugs are often used in treating sALS patients, the effects of these drugs on sALS have not been systematically studied. It is pity that long‐term antidepressant drug fluoxetine interference had no significant effect on disease progression of mutative‐SOD1 transgenic mice from postnatal 30–70 days to the final stage of disease [46]. We suggest that our evidence regarding the role of 5‑HT in the pathogenesis of ALS, along with the potential intervention strategies we propose, provides valuable insights for further in‑depth study of ALS pathogenesis and for the future exploration of intervention strategies.

Regarding the question of why 5‐HT decreases at the pre‐onset stage if the later increase is compensatory, research suggests that a decrease in the 5‐HT system function at the pre‐onset stage may represent an early dysfunction or vulnerability, potentially contributing to the development of motor neuron disease. This hypothesis is supported by our data showing a significant decrease in 5‐HT synapses in the spinal cord, especially in the AH, and diminished TPH2 expression in the brainstem's raphe obscurus at the pre‐onset stage. The raphe obscurus is a key source of descending 5‐HT projections to the spinal cord. This early loss of 5‐HT input could have several pro‐pathogenic consequences, including the loss of trophic support that deprives motor neurons of essential survival factors, making them more vulnerable to other stressors like excitotoxicity or oxidative stress present in the G93A‐SOD1 model. Additionally, reduced 5‐HT tone would remove the brake on glutamate release, potentially leading to glutamate‐induced excitotoxicity in motor neurons, a well‐established pathogenic mechanism in ALS. Furthermore, a loss of 5‐HT input would reduce motor neuron excitability by impairing the persistent inward currents crucial for sustained firing, potentially contributing to early subclinical motor dysfunction. Therefore, the pre‐onset decrease may not be a failure of compensation, but rather an early pathogenic event that creates a permissive environment for motor neuron degeneration. The subsequent increase at later stages could then be interpreted as a delayed and ultimately futile compensatory response by the surviving 5‐HT neurons, such as those in the raphe pontine nucleus, to the now‐obvious neuronal loss.

If the 5‐HT increase at onset and progression stages is not compensatory but actively pathogenic, several molecular mechanisms could be at play. 5‐HT can exert inhibitory effects through receptors such as 5‐HTR1A, while simultaneously acting as an excitatory neurotransmitter via receptors like 5‐HTR2A on motor neurons. An imbalance or dysregulation in 5‐HT release, leading to excessive levels, could potentially result in overactivation of these neurons. These excitatory receptors contribute to a hyperexcitable state and calcium overload, which ultimately accelerate motor neuron death. Our finding that 5‐HTR2A expression decreases at the onset stage in TG mice could be a protective downregulation in response to this pathological overstimulation, but this remains speculative. Additionally, reactive gliosis is a hallmark of ALS, and an abnormal increase in 5‐HT signaling might alter the communication between neurons and glia, potentially promoting the aberrant removal of synapses by microglia or astrocytes—a process contributing to neuronal dysfunction. The sustained high levels of 5‐HT could also lead to the desensitization and downregulation of key protective receptors like 5‐HTR1A, effectively short‐circuiting a neuron's own protective machinery, and the altered receptor expression we observed supports the idea of significant receptor dysregulation. Furthermore, the compensatory increase itself may turn maladaptive if the remaining 5‐HT neurons face immense stress to boost output, potentially adopting abnormal firing patterns or releasing co‐transmitters in a manner toxic to the already vulnerable motor neurons.

The interpretation of the 5‐HT increase as “compensatory protection” requires more rigorous justification. Therefore, we present three competing hypotheses rather than asserting a single protective mechanism. Hypothesis A (Compensatory protection): The increased 5‐HT at onset and progression stages may represent an attempt by surviving 5‐HT neurons to provide trophic support and modulate excitability. This is supported by the observed upregulation of 5‐HTR1A, which is known to exert neuroprotective effects via Gi/o signaling pathways, the temporal correlation with disease progression, and previous reports of 5‐HT's neurotrophic properties. Hypothesis B (maladaptive or excitotoxic response): Elevated 5‐HT could paradoxically accelerate neurodegeneration through overactivation of excitatory receptors on hyperexcitable motor neurons, facilitation of glutamate release, and induction of calcium overload. Our finding of decreased 5‐HTR2A at onset could represent compensatory downregulation against such excitotoxic stress, but this remains speculative. Hypothesis C (Epiphenomenon): The observed 5‐HT changes may be secondary to general homeostatic disruption without direct causal roles in either protection or toxicity.

Our interpretation of 5‐HTR1A and 5‐HTR2A changes must account for their distinct cellular localizations and signaling mechanisms. For 5‐HTR1A, which is Gi/o‐coupled, presynaptic localization on 5‐HT neuron terminals as autoreceptors means activation inhibits further 5‐HT release through negative feedback. Therefore, increased 5‐HTR1A protein levels could paradoxically reduce 5‐HT release despite elevated synthesis. Postsynaptic localization on motor neurons and interneurons results in hyperpolarization via GIRK channels, reducing excitability, which may be neuroprotective against excitotoxicity. Additionally, on GABAergic interneurons, 5‐HTR1A activation enhances GABA release, indirectly inhibiting motor neurons.

For 5‐HTR2A, which is Gq‐coupled, it is primarily localized postsynaptically on motor neurons and glutamatergic interneurons, where activation depolarizes neurons via TRPC channels and reduces potassium conductance, increasing excitability. On astrocytes, 5‐HTR2A activation mobilizes intracellular calcium, potentially promoting reactive astrogliosis and inflammatory cytokine release.

Thus, the same 5‐HT elevation could have opposing effects depending on which receptor population is engaged. Our finding of increased 5‐HTR1A without a parallel increase in 5‐HTR2A might suggest a shift toward inhibitory modulation, but without spatial resolution distinguishing presynaptic from postsynaptic localization, we cannot determine net circuit effects. Future studies using receptor‐selective antagonists with cell‐type‐specific knockout approaches are needed to dissect.

5‐HT's potential pathological roles (excitotoxicity): While our data show increased 5‐HT at onset and progression stages, an equally plausible interpretation is that excess 5‐HT contributes to excitotoxic motor neuron death through multiple mechanisms. First, 5‐HT can facilitate glutamate release from presynaptic terminals via 5‐HTR2A on glutamatergic afferents, potentially exacerbating the well‐established glutamate‐mediated excitotoxicity in ALS. Second, excessive 5‐HT may overactivate 5‐HTR2A on motor neurons themselves, increasing intracellular calcium via Gq‐PLC‐IP3 signaling, leading to mitochondrial dysfunction and activation of calpains and caspases. Third, sustained high 5‐HT levels could cause receptor desensitization and internalization, particularly of protective 5‐HTR1A, effectively removing a brake on motor neuron excitability. Fourth, 5‐HT may promote pathological astrocyte reactivity, as reactive astrocytes in ALS show altered calcium signaling and glutamate handling. Our observation of decreased 5‐HTR2A at onset could represent a protective downregulation against such excitotoxic stress, but this interpretation requires direct testing.

In summary, the biphasic pattern strongly suggests that the role of 5‐HT is not static. We hypothesize that the early decrease in 5‐HT levels may be a primary pathological event that contributes to disease onset, whereas the subsequent increase may represent a dysfunctional compensatory mechanism—one that is ultimately insufficient and may even exacerbate the neurodegenerative process. The observed positive correlation between elevated levels of 5‐HT, increased TPH2 distribution, and neuronal cell death suggests a significant, albeit intricate association.

Why SSRIs failed in ALS clinical trials, and why this does not invalidate our findings? Fluoxetine and other selective serotonin reuptake inhibitors have failed to show therapeutic benefit in ALS clinical trials and animal models. This apparent contradiction requires explicit explanation, as it directly challenges the translational relevance of our findings.

The critical distinction is receptor selectivity. SSRIs non‐selectively elevate extracellular serotonin throughout the central nervous system by blocking the serotonin transporter. This global elevation activates all serotonin receptor subtypes simultaneously. Our data reveal that, in G93A‐SOD1 mice at the onset stage, two major serotonin receptors show opposing regulation: 5‐HTR1A is significantly upregulated while 5‐HTR2A is significantly downregulated. These two receptors mediate opposing effects on motor neurons. 5‐HTR1A is an inhibitory Gi‐coupled receptor that reduces neuronal excitability and protects against excitotoxicity. 5‐HTR2A is an excitatory Gq‐coupled receptor that facilitates glutamate‐induced calcium overload and promotes excitotoxic stress. An SSRI would indiscriminately activate both receptors, potentially canceling out any protective effect from 5‐HTR1A activation while simultaneously stimulating the already downregulated 5‐HTR2A system. This provides a mechanistic explanation for SSRI failure that does not contradict our finding that the endogenous serotonin system shows a selective, potentially compensatory response.

Additional reasons for SSRI failure as follows: Chronic SSRI administration leads to desensitization of 5‐HTR1A autoreceptors on raphe neurons, which paradoxically reduces serotonin neuron firing after an initial transient increase. SSRIs also fail to address the pre‐onset decrease in serotonin synapses that we observed in the anterior horn of the spinal cord, which may represent the critical therapeutic window. Furthermore, SSRIs affect peripheral serotonin stored in platelets, where serotonin levels paradoxically predict survival in ALS patients, suggesting that central and peripheral serotonin compartments have opposing relationships with disease progression.

### 4.1 Specific Translational Strategies

Based on our receptor expression data, we propose three specific, mechanistically distinct intervention strategies that differ fundamentally from failed SSRI approaches.

First, 5‐HTR1A agonism represents the most promising strategy. Our finding that 5‐HTR1A is significantly upregulated at the onset stage suggests that the endogenous serotonergic system is attempting to enhance inhibitory tone onto motor neurons. This protective attempt could be pharmacologically augmented using existing FDA‐approved drugs. Buspirone, an FDA‐approved anxiolytic that acts as a 5‐HTR1A partial agonist, has a favorable safety profile and crosses the blood–brain barrier. It could be repurposed for ALS at low, intermittent doses to avoid autoreceptor desensitization. For preclinical proof of concept, the research tool 8‐OH‐DPAT or the highly selective repinotan (which completed stroke trials) could be tested in G93A mice. The testable hypothesis is that 5‐HTR1A agonism will recapitulate the compensatory increase pattern we observed at the progression stage and slow motor neuron death.

Second, 5‐HTR2A antagonism offers an alternative or complementary approach. We observed that 5‐HTR2A significantly decreases at the onset stage, which may represent a protective downregulation to reduce excitotoxic signaling. Pharmacological antagonism could mimic or enhance this effect. Low‐dose risperidone, an FDA‐approved antipsychotic with potent 5‐HTR2A antagonist activity, could be repurposed at doses below those causing significant dopamine receptor blockade. The research tool ketanserin provides a more selective option for preclinical studies. The hypothesis is that 5‐HTR2A blockade will reduce glutamate‐induced excitotoxicity in motor neurons.

Third, precursor loading with receptor targeting may address the pre‐onset deficit. Turner and colleagues reported in 2003 that the serotonin precursor 5‐hydroxytryptophan delayed disease onset in G93A mice, but this study has been under‐cited and its mechanistic basis is unclear. Our data provide mechanistic support for this observation. However, to avoid non‐specific receptor activation, we propose combining 5‐hydroxytryptophan with carbidopa (to prevent peripheral conversion) plus a 5‐HTR1A agonist to channel the newly synthesized serotonin toward protective receptors.

Why These Strategies Differ From Failed SSRIs? The fundamental difference is selectivity. SSRIs are like using a sledgehammer; they elevate serotonin everywhere and activate every receptor. Our proposed strategies are like using a scalpel; they target specific receptors that our data show are differentially regulated in the diseased state. The opposing regulation of 5‐HTR1A and 5‐HTR2A that we discovered provides a clear mechanistic rationale for why non‐selective approaches failed and why selective approaches might succeed. This is analogous to the evolution of dopamine therapy in Parkinson's disease: non‐specific dopamine precursors had limited benefit, but selective dopamine receptor agonists became standard of care.

Several clinical trials have examined SSRI antidepressants in ALS patients and animal models, with largely negative results. Our findings do not contradict these reports but rather provide a mechanistic explanation for them. SSRIs non‐selectively elevate extracellular serotonin, activating both protective 5‐HTR1A and potentially deleterious 5‐HTR2A receptors. In contrast, our data revealed opposing regulation of these receptor subtypes in TG mice, with 5‐HTR1A upregulated and 5‐HTR2A downregulated at the onset stage. This suggests that the endogenous serotonergic response is not merely quantitative but qualitatively selective. Therefore, we propose that successful serotonin‐based therapy requires receptor‐selective approaches: 5‐HTR1A agonism, 5‐HTR2A antagonism, or combined precursor loading with receptor targeting, rather than global serotonin elevation.

We acknowledge that these proposed strategies remain hypothetical and require rigorous preclinical testing in G93A mice before clinical consideration. The negative SSRI trials indicate that non‐selective serotonin modulation is unlikely to succeed, but they do not preclude the potential efficacy of receptor‐selective approaches guided by the differential receptor regulation we have discovered.

## 5 Limitations and Prospects

This study has several limitations. First, the immunohistochemical approach is unable to determine whether increased 5‐HT and TPH2 distribution stems from enhanced synthesis or impaired release, leaving the functional significance remains unclear. Second, receptor analysis was limited to only 5‐HTR1A and 5‐HTR2A, excluding other receptor subtypes that could potentially influence motor neuron function. Third, the correlational findings do not establish causality between 5‐HT alterations and neuronal death. Fourth, the study used only the G93A‐SOD1 mouse model, which may not be generalizable to other forms of ALS. Fifth, neurochemical changes were not found to be directly correlated with behavioral deficits. Sixth, the three time points examined may miss critical alterations during disease transition. Seventh, synaptic localization of 5‐HT changes was inferred rather than directly confirmed. Moreover, peripheral 5‐HT system alterations were not examined. Seventh, this study only used male mice, there were obvious gender differences in ALS, and the prognosis of female patients was usually poor. The 5‐HT system is significantly regulated by sex hormones, and the extrapolation of single‐sex results is limited. Therefore, we outline plans for follow‐up studies comparing both sexes, including ovariectomized females with hormone replacement or estrous cycle staging, to isolate the contribution of sex hormones to 5‐HT system function in ALS.

Current findings indicate a significant accumulation of 5‐HT, which could be attributed to enhanced synthesis or reduced release/reuptake. The precise mechanism, however, remains to be elucidated. To address this, we propose two lines of investigation. Initially, we will assess the levels of 5‐Hydroxyindoleacetic acid (5‐HIAA), the primary metabolite of 5‐HT, to understand its role in physiological processes and as a diagnostic marker. An increase in both 5‐HT and 5‐HIAA levels suggests enhanced synthesis and turnover. Conversely, unchanged or decreased levels of 5‐HIAA could indicate a deficit in release or clearance. Second, we will analyze serotonin transporter (SERT) expression; a decrease in SERT activity would suggest that impaired 5‐HT reuptake is responsible for the observed accumulation of 5‐HT. These experiments will clarify the mechanistic basis of our observations and guide future therapeutic strategies.

A particularly notable finding of our study is that, in the spinal cord of TG mice, a differential regulation of 5‐HT receptor subtypes has been observed, with a significant increase in 5‐HTR1A protein levels at the disease stage, while 5‐HTR2A expression remained stable or exhibited a downward trend. The functional divergence of these receptors is significant because they mediate distinct effects, which in the context of motor neuron function can be opposing. Research has shown that 5‐HT and its receptors play a crucial role in motor diseases, influencing fatigue recovery and motor neuron function. 5‐HTR1A is predominantly an inhibitory Gi/o‐protein‐coupled receptor whose activation on motor neurons typically leads to neuronal hyperpolarization and a reduction in excitability, and it is known to exert neuroprotective effects in various models of neurodegeneration by reducing excitotoxicity and promoting cell survival. Conversely, 5‐HTR2A is a Gq‐protein‐coupled receptor that is often excitatory, and its activation on motor neurons can facilitate glutamate‐induced depolarization and increase intracellular calcium, potentially contributing to excitotoxic stress, a well‐established pathogenic mechanism in ALS. Therefore, the selective upregulation of 5‐HTR1A in the spinal cord of TG mice, coupled with the absence of a parallel increase in the more excitatory 5‐HTR2A, may represent a specific and targeted compensatory response. As the disease progresses and motor neurons become increasingly vulnerable, surviving 5‐HT neurons—or the motor neurons themselves—may attempt to limit excitotoxic damage by enhancing inhibitory signaling through the 5‐HT1A receptor. At the same time, they may engage other protective mechanisms, such as those involving iron homeostasis and the mitigation of oxidative stress, as suggested by ALS pathology studies. This adaptive response appears to avoid a concurrent increase in excitatory 5‐HT2A receptor signaling, which could otherwise worsen neuronal stress. Our findings of elevated spinal cord 5‐HT levels are consistent with the notion that the system may not only be increasing its output but also potentially modulating receptor activation to a more protective state, as evidenced by the role of 5‐HT in spinal pain modulation and Gamma‐aminobutyric acid synthesis enhancement.

To address the lack of direct evidence of “compensatory protection” mechanism, future studies should employ conditional 5‐HT neuron ablation or chemogenetic silencing in TG mice to determine whether 5‐HT elevation is necessary for survival, pharmacological manipulation using 5‐HTR1A antagonists and 5‐HTR2A agonists to test causality, in vivo microdialysis to measure real‐time 5‐HT release correlated with motor neuron activity, and longitudinal assessment of 5‐HT turnover via 5‐HIAA to 5‐HT ratios to distinguish enhanced synthesis from impaired clearance.

We did not test any pharmacological intervention in this study; our correlational findings cannot establish causality or directly predict therapeutic efficacy. The failure of SSRIs in ALS models underscores that non‐selective serotonin elevation is insufficient, and our proposed receptor‐selective strategies must be validated by interventional studies before any clinical translation can be considered.

## 6 Conclusion

In general, our study suggested that 5‐HT at the pre‐onset stage, the number of synapses in the spinal cord, and 5‐HT neurons in the brainstem significantly decreased; however, at the onset and/or progression stage, the number of 5‐HT synapses and neurons significantly increased. This study also suggested that non‐MN 5‐HT neurons played a role in the pathogenesis of ALS by releasing 5‐HT neurotransmitter into the projecting neuron, as supported by research on neurotransmitter chemistry and the role of these neurons in CNS synapses, and that 5‐HT neuron involved the damage course of MN in the cortical and subcortical regions as well as the nuclei of brain stem and the AH of spinal cord was a potential protective pathogenesis of neuron death in G93A‐SOD1 TG mice.

## Author Contributions

L.j.Z., M.h.L., Q.D., X.w.L., H.f.J., and C.L. performed experiments, analyzed the data, and wrote the manuscript; L.j.Z., M.h.L., and C.L. conducted statistical analyses; L.j.Z., and M.h.L. were the common jointed authors and equally contributed to this study. R.s.X. conceived the project and wrote the manuscript. L.j.Z., R.s.X., and H.l.P. revised the manuscript. All authors read and approved the final manuscript.

## Funding

The authors received no financial support for the research, authorship, and/or publication of this article. This study was supported by the research grants from the National Natural Science Foundation of China (30560042, 81160161, 81360198, and 82160255), Education Department of Jiangxi Province (GJJ13198 and GJJ170021), Jiangxi Provincial Department of Science and Technology (20142BBG70062, 20171BAB215022, 20192BAB205043, and 20212BAB216026), Health and Family Planning Commission of Jiangxi Province (20181019, 202110016, and 202310119), Jiangxi Province Key Laboratory of Neurology (2024SSY06081), Science and Technology Plan Project of Jiangxi Provincial Administration of Traditional Chinese Medicine (2024A0159), and Key Research and Development Project of Jiangxi Provincial Department of Science and Technology (20243BBI91030).

## Ethics Statement

All animal studies and experiments were conducted in accordance with the Guide for the Care and Use of Laboratory Animals and were reviewed and approved by the ethics committee for animal care and use of the First Affiliated Hospital of Nanchang University, China. All procedures in this study were conducted in accordance with the ethics committee for animal care and use of the First Affiliated Hospital of Nanchang University, China (Ethic approval number: 2015/3‐12‐K).

## Consent

All authors have read and approved the content and agree to submit for consideration for publication in the journal.

## Conflicts of Interest

The authors declare no conflicts of interest.

## Supporting information


**Data S1:** Supporting Information

## Data Availability

The original contributions presented in the study are included in the article/Supporting Information; further inquiries can be directed to the corresponding author/s.
