# Supplementary figures and images for: 5‐Hydroxytryptamine Distribution Alteration in Both Neuron and Synapse of Tg(SOD1*G93A)1gur Mice: A Potential Intervention Candidate Strategy for Amyotrophic Lateral Sclerosis
Source: CNS Neurosci Ther. 2026 May 29;32(6):e70946. doi: 10.1002/cns.70946 (PMC13240577; doi:10.1002/cns.70946)

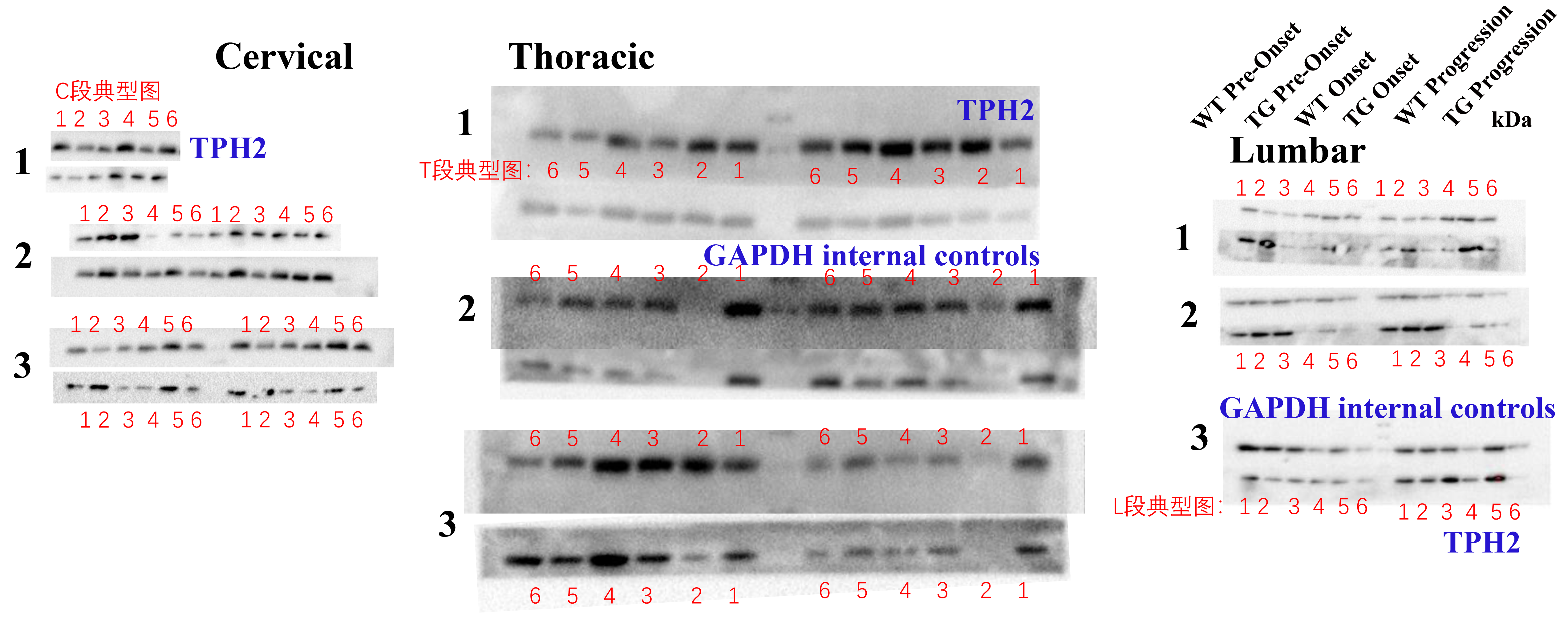

Supplement: Supplementary file 1 — Data S1: Supporting Information [file CNS-32-e70946-s001.zip › Supplementary files/TPH2-Western blot raw data.tif]
